# Successional dynamics and alternative stable states in a saline activated sludge microbial community over 9 years

**DOI:** 10.1186/s40168-021-01151-5

**Published:** 2021-10-06

**Authors:** Yulin Wang, Jun Ye, Feng Ju, Lei Liu, Joel A. Boyd, Yu Deng, Donovan H. Parks, Xiaotao Jiang, Xiaole Yin, Ben J. Woodcroft, Gene W. Tyson, Philip Hugenholtz, Martin F. Polz, Tong Zhang

**Affiliations:** 1 Environmental Microbiome Engineering and Biotechnology Laboratory, The University of Hong Kong, Hong Kong SAR, China; 2 Australian Centre for Ecogenomics, School of Chemistry and Molecular Biosciences, The University of Queensland, Brisbane, QLD Australia; 3 School of Engineering, Westlake University, 18 Shilongshan Road, Hangzhou, 310024 China; 4 Department of Civil and Environmental Engineering, Massachusetts Institute of Technology, Cambridge, MA 02139 USA; 5 Division of Microbial Ecology, Centre for Microbiology and Environmental Systems Science, University of Vienna, Vienna, Austria

**Keywords:** Microbial community, Alternative stable states, Time-series, Activated sludge, Disturbance, Stability, Resilience

## Abstract

**Background:**

Microbial communities in both natural and applied settings reliably carry out myriads of functions, yet how stable these taxonomically diverse assemblages can be and what causes them to transition between states remains poorly understood. We studied monthly activated sludge (AS) samples collected over 9 years from a full-scale wastewater treatment plant to answer how complex AS communities evolve in the long term and how the community functions change when there is a disturbance in operational parameters.

**Results:**

Here, we show that a microbial community in activated sludge (AS) system fluctuated around a stable average for 3 years but was then abruptly pushed into an alternative stable state by a simple transient disturbance (bleaching). While the taxonomic composition rapidly turned into a new state following the disturbance, the metabolic profile of the community and system performance remained remarkably stable. A total of 920 metagenome-assembled genomes (MAGs), representing approximately 70% of the community in the studied AS ecosystem, were recovered from the 97 monthly AS metagenomes. Comparative genomic analysis revealed an increased ability to aggregate in the cohorts of MAGs with correlated dynamics that are dominant after the bleaching event. Fine-scale analysis of dynamics also revealed cohorts that dominated during different periods and showed successional dynamics on seasonal and longer time scales due to temperature fluctuation and gradual changes in mean residence time in the reactor, respectively.

**Conclusions:**

Our work highlights that communities can assume different stable states under highly similar environmental conditions and that a specific disturbance threshold may lead to a rapid shift in community composition.

Video Abstract

**Supplementary Information:**

The online version contains supplementary material available at 10.1186/s40168-021-01151-5.

## Introduction

Because humans rely on myriads of services provided by complex microbial communities in both natural and applied settings, an important question is how environmental change affects community structure and function. The classical view from macroecology is that communities display resilience toward perturbation until a tipping point is reached after which the system may enter a new state. Such alternative states can be stable and have different properties from the original [1]. For microbial systems, recent work has suggested that communities of different taxonomic composition can encode much of the same metabolic pathways, and hence be functionally highly similar [2, 3]. However, it remains poorly understood how stable these taxonomically diverse assemblages can be and what causes them to transition between states.

Activated sludge (AS) is a prime example of complex microbial communities mediating important engineered functions. These communities self-assemble according to the operational parameters of wastewater treatment plants (WWTPs) [4] and affect the removal of pollutants, including organic carbon, nitrogen, and phosphorus, from wastewater [5, 6] and prevent the spread of wastewater-borne diseases [7, 8]. A key question thus is how reliably these processes occur under regular variation of WWTP operational parameters as well as under more irregular disturbances. While high throughput amplicon sequencing of the 16S rRNA gene has been widely applied to resolve the complex microbial community structure in AS at different temporal and spatial scales [9–17], the relationship between community dynamics and metabolic functions cannot be unraveled by taxonomic surveys alone. Some statistical analyses have suggested deterministic community assembly mediated by environmental filtering and biological interactions in AS [18–22], but it remains unknown how complex AS communities evolve in the long term, and how the community function changes when there is a disturbance in operational parameters.

With advances in sequencing technology and computing resources, numerous bacterial and archaeal metagenome-assembled genomes (MAGs) recovered from AS metagenomes have greatly expanded our understanding of conserved and shared functional guilds in full-scale AS systems [23, 24]. Despite the large-scale spatial studies that can be used to construct the genome catalog of microorganisms in AS systems, single or limited sampling points cannot unravel ecological insights on community dynamics and response to disturbance [25]. Here, we apply metagenomic sequencing to 97 monthly AS samples over 9 years (June 2007 to December 2015) from the same aeration tank of Shatin WWTP (Hong Kong SAR, China). To characterize the community composition and temporal dynamics, we recover non-redundant bacterial and archaeal MAGs that represent the majority of the community in the AS ecosystem. We show that one transient and simple disturbance can abruptly change the taxonomic composition to an alternative state. Using the reconstructed gene catalog of the AS system, we determine the impact of microbial community turnover on the changes of metabolic functions relating to the performance of WWTP. We also use network inference to link the microbial cohorts with correlated dynamic patterns to the regular seasonal variation and the onset of gradual operational changes of the WWTP. We further conduct comparative genomic analyses and reveal the metabolic differentiation of microbial cohorts in response to the changes in operational parameters. Overall, our work suggests that under highly similar conditions, alternative stable community states may exist and be characterized by different taxonomic composition but similar functional capabilities.

## Methods

### AS sampling and environmental data

AS samples were collected monthly from June 2007 to December 2015 (Supplementary Table S1) at the same sampling point in Shatin WWTP (22.406 N, 114.213 E), which is the largest secondary WWTP treating saline sewage in Hong Kong. As approximately 80% of the residents are supplied with seawater for toilet flushing, the sewage treated by Shatin WWTP is saline wastewater comprising ∼30% of seawater. Samples were diluted with absolute ethanol at a volume ratio of 1:1 for biomass fixation and then stored in a refrigerator at −20 °C. Operational parameters were collected from the Drainage Services Department, Hong Kong. As the studied WWTP encountered a seasonal foaming problem, engineers started small trial experiments at the end of December 2009 and full trial experiments (1–3 g Cl_2_/kg MLSS/day) since February 2010 by adding bleach solution (sodium hypochlorite). The bleach solution was continuously added to the aeration tanks from 1 January 2011 to 31 May 2011, while batch addition was adopted in other months with bleaching events. The addition of bleach solution effectively alleviated the biological foaming and stopped in 2014. Other detailed information regarding sewage treatment performance, physicochemical conditions are summarized in Supplementary Table S2.

### DNA extraction and metagenomic sequencing

A 1 mL aliquot of each diluted sample was centrifuged to obtain a pellet of ~ 200 mg, which was subject to DNA extraction with the FastDNA Spin Kit for Soil (MP Biomedicals). The extracted DNA samples were then sequenced on an Illumina HiSeq 4000 platform (150 bp paired-end reads, 350 bp insert size) by the Beijing Genomics Institute (BGI), generating a total of 97 metagenome datasets with sequencing amounts 5.6 ± 0.76 Gb. A total of 539.9 Gb metagenomic sequencing data were generated from the time series AS samples. We have previously shed light on the dynamics of virome [26] and antibiotic resistance genes (ARGs) [27].

### Gene catalog and MAGs recovery

Quality controlled reads were de novo assembled using CLC Genomic Workbench (version 6.04, QIAGEN Bioinformatics, Denmark) and MEGAHIT [28]. Different assembly strategies, including single dataset assembly and multiple samples co-assembly, were used in the present study. Briefly, metagenomic sequences from AS samples were independently assembled and co-assembled (yearly samples) using CLC Genomic Workbench with a *k*-mer of 35, automatic bubble size, and scaffolding. The assembled scaffolds with length > 1 kb were retained for downstream analysis (Supplementary Table S1). Also, metagenomic sequences of all AS samples were co-assembled using MEGAHIT with default parameters to recover the contigs that were not assembled due to limited sequencing depth. The open reading frames (ORFs) on all the assembled contigs were predicted using Prodigal (-meta mode) (v2.6.3) [29] and clustered using CD-HIT (v4.6.8) [30] at a cutoff of 95% sequence identity and 90% alignment coverage of the shorter sequence [31]. We obtained a gene catalog comprising 12,588,406 non-redundant genes. Rarefaction curve of detected genes in given number of AS samples was summarized using a python script (Supplementary S1, script 1). To taxonomically annotate the recovered gene catalog, the non-redundant genes were searched against NCBI nr database (release March 24, 2019) using DIAMOND v0.9.22.123 [32] with *E* value ≤ 1e − 5. Reference protein ids of best hits were used to parse the taxonomic affiliation.

The co-assembly results of each year were imported to MetaWRAP to recover bacterial and archaeal MAGs. Both the bin_refinement and reassembly_bins modules from MetaWRAP were performed to refine the recovered MAGs and improve completion and N50 of the newly recovered MAGs, respectively. The completeness and contamination of the newly recovered MAGs were estimated using CheckM (v1.0.18) [33] with lineage-specific marker genes with default parameters (Supplementary Table S3). Only MAGs with completeness ≥ 50% and contamination ≤ 10% were retained for downstream analysis. The recovered MAGs from different years’ samples were dereplicated using dRep (v2.3.2) [34] at the thresholds of 90% Mash similarity for the primary clustering and 99% ANI for the secondary clustering (default parameter), ≥ 50% completeness, and ≤ 10% contamination to remove the replicated MAGs.

### Phylogenomic tree

Sets of 122 archaeal and 120 bacterial marker genes from the newly recovered MAGs and the selected reference genomes close to the newly recovered MAGs (NCBI RefSeq database) were classified using the ‘identify’ module of GTDB-Tk (v0.2.1) [35, 36]. The identified marker genes were then aligned and concatenated using the align module of GTDB-Tk [36]. FastTree (v2.1.10) [37] was then used to infer a genome tree based on the concatenated alignment of the identified markers genes under the WAG + GAMMA model [36]. The genome tree was imported into iTOL [38] for further refinements.

### Genome annotation

Genes in the newly recovered MAGs were predicted using Prodigal (v2.6.3) [29] and initially annotated using Prokka (version 1.11) [39]. The genes in each MAG were also searched against the KEGG prokaryotes database (release 80.1) using DIAMOND (v0.9.22.123) [32], taking the best hit with an *E* value < 1e − 10 to assign the predicted genes to KEGG orthology (KO) groups. Metabolic pathways in MAGs were then reconstructed using KEGG Mapper (v4.1) [40]. The MAGs that need further investigations were annotated using online platforms GhostKOALA [41] and IMG/MER [42]. All protein sequences from each MAG were searched against dbCAN HMMs V7 [43, 44] using HMMSCAN [45]. The hmmer3 outputs were parsed by the hmmscan-parser.sh script. The diversity and abundance of CAZy modules identified in each MAG were summarized using an online script (https://github.com/yuboer/genome-centric-portrait-of-cellulose-hydrolysis). The potential transporter genes of studied MAGs were performed using the Transporter Automatic Annotation Pipeline (TransAAP) (membranetransport.org).

### Dynamics of microbial community and functional structure

Metagenomic sequences and MAGs based microbial community dynamics were conducted in this study. Metagenomic sequences from each sample were assigned to taxonomic labels using three different approaches: Kraken (v2.0.7) [46], mOTUs2 (v2.1.0) [47], and SingleM (v0.12.0, https://github.com/wwood/singlem). For Kraken and mOTUs2, the default parameters were used to profile the community structure. For SingleM, the relative abundance of community structure was estimated based on the sequence count of ribosomal protein L2. The relative abundance of the dereplicated MAGs was also calculated using CoverM (v0.2.0, https://github.com/wwood/CoverM), which mapped metagenomic sequences to the MAGs with default parameters. The microbial community composition was summarized based on the taxonomic assignment at phylum level. The community dynamics of sequences were compared to MAGs-based results.

Vegan [48] was used to calculate the alpha- and beta-diversity based on the microbial community composition results of SingleM and MAGs-based analyses. Principal coordinates analysis (PCoA) of microbial community composition was conducted based on the Bray–Curtis distance. Distance-based redundancy analysis (dbRDA) [49] was used to find the environmental variables that best explained the patterns of community dynamics over time. Significant variables were determined with a multivariate non-parametric ANOVA (Benjamini–Hochberg adj. *P* < 0.01). Additionally, a time-decay dissimilarity analysis between AS samples was conducted to explore the recurrence of the AS microbial communities (Supplementary S1, script 2).

To profile the dynamics of functional structure of the microbial community, the non-redundant gene catalog was annotated to KOs following the method mentioned above. The coverage of functional genes in each sample was calculated using CoverM at a cutoff of 95% sequence identity and 50% alignment coverage of the mapped metagenomic sequences. For each functional pathway, the abundance was calculated as the sum of marker KOs’ coverage normalized by the number of KOs. For the comparative analyses between AS samples, the abundance of a given functional pathway was normalized by the sums of all studied pathways’ abundance.

### Network analysis and microbial community clustering

To investigate the time-dependent correlations between microbial members and environmental variables, the extended local similarity analysis (ELSA, v1.0.6) [50] was used for network analysis. The *P* value estimation was calculated with theoretical approximation (-p theo). Dynamics of bacterial and archaeal members that have been identified as relatively abundant populations (relative abundance > 0.5%) in at least one AS sample were integrated with environmental variables for network analysis. The strong (local similarity (LS) score ≥ 0.6 or ≤  − 0.6) and statistically significant (*P* value ≤ 0.05; False-discovery rate, that is *Q* value, ≤ 0.01) correlations were retained from the eLSA analysis results. Synchronous associations and time-shifted (1 month) correlations between microbes and between microbe and environmental factors were visualized using Cytoscape v3.7.1 [51]. Statistical and topological characteristics of the network were determined for undirected networks using Network Analyzer [52]. Microbial community clustering was performed using the Markov CLustering Algorithm (MCL) in the Cytoscape plugin clusterMaker [53] based on the values of LS, which generated five clusters (C1-C5). Besides, the relative abundance values of MAGs used for network analysis were normalized by *Z* score. The normalized matrix is then used to perform partitional clustering based on the *K*-medoids algorithm (pam function from R package ‘cluster’). The number of clusters (*k*) for *K*-medoids analysis was determined by the Davies-Bouldin index [54].

### Statistics for metabolic traits of microbial community

EnrichM v0.5.0 (https://github.com/geronimp/enrichM) was used to identify the statistically enriched KEGG modules between classified microbial cohorts. Briefly, all functional genes in MAGs from different microbial cohorts were annotated with KO. The obtained KO frequency matrix for annotated MAGs was used to perform statistical tests for the enrichments of KEGG modules between any two identified microbial cohorts. Pairwise Mann–Whitney *U* test was applied to the number of significantly enriched steps (i.e., reactions) present between MAGs from microbial cohorts [55]. Only enriched modules with > 60% completeness (enriched steps/all steps for a metabolic module) were discussed in the present microbial cohorts pairwise comparison.

## Results

### Gene and genome catalog of activated sludge microbiome

From our longitudinal sampling of AS microbiota over 9 years, we recovered 539.9 Gb DNA sequence data from 97 samples with an average (± standard deviation) data size of 5.6 (± 0.76) Gb (Supplementary Table S1). To better explore the genetic diversity, we first constructed a reference gene catalog for the AS system by predicting ORFs from assembled contigs, recovering 12.6 M (million) non-redundant genes from bacteria (73.2%), eukaryotes (1.4%), archaea (0.6%), viruses (0.2%), and unclassified taxa (24.6%). This gene catalog recruited an average of 82.4% (± 5.0) of metagenomic sequence reads from the samples (Supplementary Table S4). Rarefaction analysis showed asymptotic behavior, suggesting that the major genes were nearly completely sampled when the sample number was more than 55 and sequencing depth more than ~ 300 Gb (Supplementary Figure S1).

**Fig. 1:**
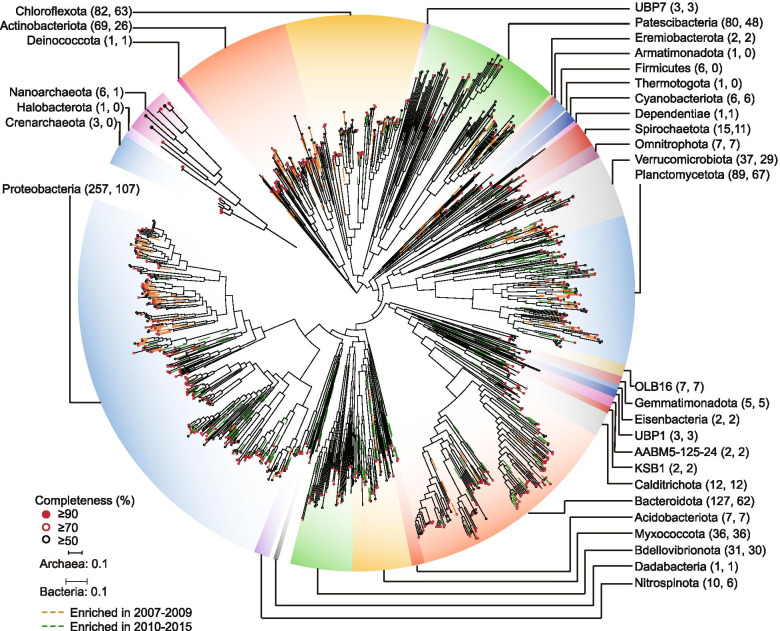
Phylogenetic distribution of the 920 dereplicated MAGs recovered in the present study and selected reference genomes. The phylogenetic tree was inferred from 120 bacterial and 122 archaeal proteins. The identification and alignment of the conserved proteins were performed using the GTDB-Tk [56]. The completeness of these MAGs was estimated using CheckM [33]. The branches of MAGs that significantly enriched in the first 3 years’ sample and the following 6 years are colored in orange and green. The numbers in brackets represent the recovered MAGs and MAGs cannot be assigned into reported genera

We further recovered 2178 MAGs with completeness ≥ 50% and contamination ≤ 10% from the co-assembled metagenomes. After dereplication at an average nucleotide identity (ANI) cutoff of 99% [55], 920 MAGs were retained and used for further genome-centric analysis (Supplementary Tables S4). These MAG clusters recruited an average of 69.7% (± 2.6) of the metagenomic reads from different AS samples (Supplementary Table S5). Based on the phylogenomic tree (Fig. 1), most of these MAGs were affiliated with the bacterial phyla Proteobacteria (257 MAGs), Bacteroidota (127), Planctomycetota (89), Chloroflexota (82), Patescibacteria (80), Actinobacteriota (69), Verrucomicrobiota (37), Myxococcota (36), and Bdellovibrionota (31). There were also MAGs from the candidate phyla Calditrichota (12 MAGs), OLB16 (7), Nanoarchaeota (6), UBP1 (3), UBP7 (3), AABM5 (2), Delongbacteria (2), Eremiobacterota (2), KSB1 (2), SAR324 (1), and UBP3 (1). Notably, approximately 60% of the recovered MAGs were novel and affiliated with unclassified genera based on the Genome Taxonomy Database [36, 56] (Supplementary Table S3), indicating that the saline activated sludge under study is a reservoir of yet-to-be-cultured and functionally uncharacterized populations.

### Simple disturbance induced alternative stable state

**Fig. 2:**
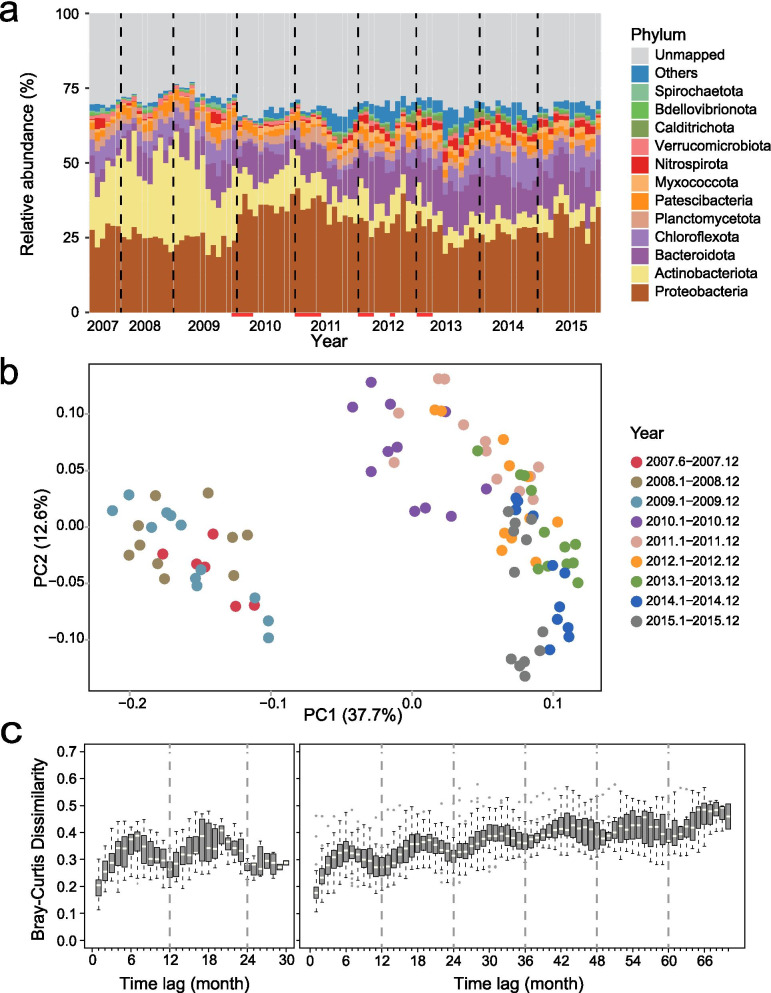
Dynamics of microbial community and microbial diversity. **a** Phylum-level taxonomic variability shows the relative abundance based on the ratio of recruited reads of the dereplicated MAGs. Bleaching events are marked with red lines on the *x*-axis. **b** Principal coordinate analysis (PCoA) of studied AS samples based on Bray–Curtis distances of MAG clusters-based community structure. **c** Time lag between samples (first 3 years and following 6 years) versus Bray–Curtis dissimilarity. Only MAGs represented in at least 20% of samples are used for establishing community dynamics

The community in the AS system displayed remarkably high temporal stability that was, however, pushed to an alternative stable state by a single disturbance after 3 years (Fig. 2a and b). The disturbance was caused by the addition of bleach solution (NaOCl) at the end of 2009 to control biological foaming (Supplementary Table S2). This intervention led to a major community shift from an Actinobacteriota to a Proteobacteria dominated community both of which were stable over many months, hence, the designation as an alternative stable state [57]. Specifically, 50.7% of bacteria within the phylum Actinobacteriota (35 MAGs) and 57.3% of bacteria within the class Gammaproteobacteria (71 MAGs) were significantly (adj. *P* < 0.05) enriched in the samples before and after the end of 2009, respectively (Fig. 1 and Supplementary Table S3). A recent study reported the global AS communities found that most of the core bacterial members belonged to Proteobacteria [17], which was consistent with the observed dominance of proteobacterial organisms after bleaching event. While the AS community dominated by taxa within the phylum Actinobacteriota was not observed in that global AS sampling campaign [17]. Moreover, the decline of bacteria was accompanied by emergence of new bacteria with distinct phylogenomic affiliations after the end of 2009 (Supplementary Figure S2), demonstrating the change of taxonomic composition after the bleaching event. This sharp change in microbial community composition was further confirmed by read-based diversity analysis (Supplementary Figures S3, S4 and Table S6). Despite the fact that bleach solution was also used in the Spring of 2011 to 2013, this did not lead to a similarly dramatic change in microbial community composition, indicating that the community had changed to a stable state that was more resilient to this periodic stressor.

Although the two community states before and after the primary bleaching event appeared stable, the MAG-based community profiles showed some changes in Bray–Curtis similarity that consisted of (i) seasonal fluctuations with a periodicity of ~ 12 months, and (ii) a moderate decay over time (Fig. 2c). While the seasonal fluctuations might be driven by seasonal temperature variation, community changes over longer periods showed a close relationship with the increase in mean cell residence time (MCRT). Distance-based redundancy analysis (Supplementary Figure S5 and Table S7) revealed that these two factors together explained 22.5% of the compositional variation, while MCRT showed a relatively strong correlation with PC1 (*R*^2^ = 0.45; Supplementary Figure S6). The MCRT increased after the middle of 2010 and was associated with a gradual increase in richness of microbial communities (Supplementary Figure S7). Moreover, warmer temperature appeared to be associated with higher diversity since one-way ANOVA of Pielou evenness and Shannon index revealed a seasonal trend with the highest (Mann–Whitney, *P* < 0.05) alpha-diversity being identified in summer. These analyses thus suggested that increase in MCRT led to gradually increasing diversity in the community state post disturbance, while the seasonal fluctuations in diversity persisted across the disturbance.

### Succession of microbial cohorts underpins taxonomic turnover

**Fig. 3:**
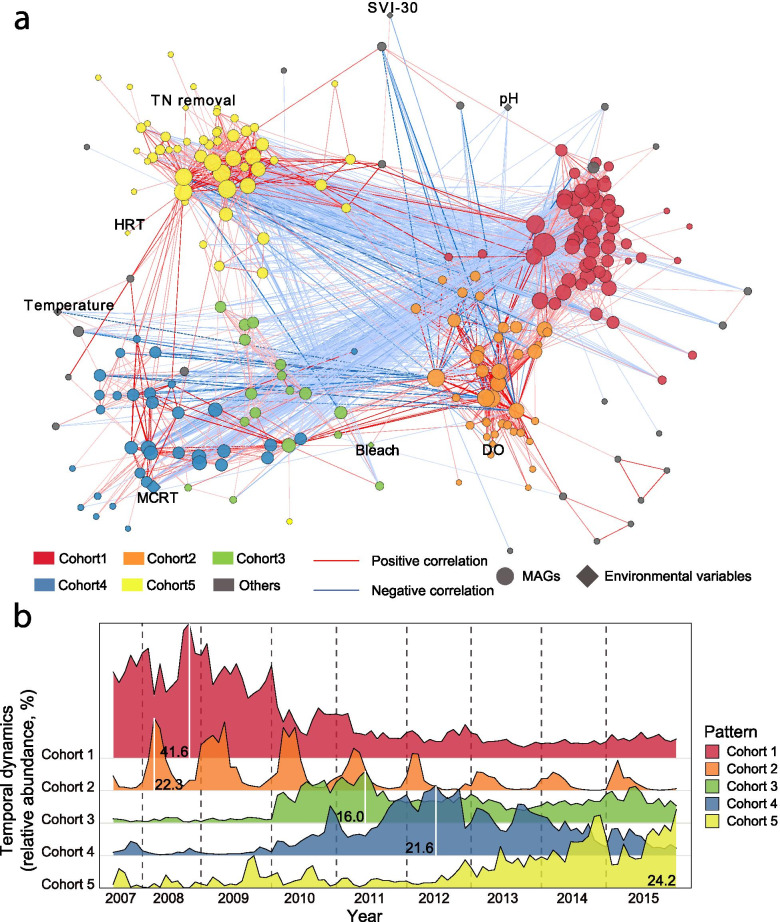
Identification of microbial cohorts. **a** Environment-MAGs network uncovered local and potentially time-delayed co-occurrence and association patterns between bacteria and environmental variables in activated sludge. Only statistically significant (*P* value ≤ 0.05, *Q* value ≤ 0.01) and strong (local similarity score ≥ 0.6 or ≤  − 0.6) correlations are shown in this figure. The line thickness is proportional to the absolute value of local similarity, and dash line indicates a 1-month shift/delay in the correlation. Node size and color represent the degree and identified clusters using Markov CLustering Algorithm (MCL). **b** Ridge plot shows the temporal dynamics of identified microbial cohorts. The numbers in the ridge plot are the maximum relative abundance of microbial cohorts

Further analysis of the observed seasonal and longer term community turnover revealed that changes in taxonomic richness are due to successions of defined cohorts (clusters of correlated MAGs). We analyzed MAGs that showed a relative abundance > 0.5% at least once in the time series to investigate whether they shifted in a correlated manner within the overall community [58]. Based on this analysis, these more abundant organisms were grouped into five distinct cohorts (C1-C5) based on local similarities calculated by extended Local Similarity Analysis (eLSA) [50] (Fig. 3a). Four of these cohorts were confirmed by *K*-medoids-based partitioning clustering analysis based on the Davies-Bouldin index (DBI) (Supplementary Figure S8). Most of the MAGs within C1 (96.7%), C2 (82.1%), C4 (84.8%), and C5 (91.8%) were consistent among the two methods, while taxa within C3 were not resolved by *K*-medoids-based analysis due to the prespecified number of clusters (*K*).

The cohorts showed cohesive dynamics over different time periods suggesting that their members respond differently to seasonal and operational factors of the treatment plant. Cohort 1 (C1) dominated in the first 3 years (2007–2009) prior to the disturbance and increase in MCRT, accounting for an average of 41.6% of the total metagenomic sequence reads. After the primary bleaching event, successional dynamics occurred with 3 cohorts partially replacing each other after a few years. Cohort C3 dominated in the 2 years after the bleaching event (2010–2011), followed by cohorts C4 (2012–2013) and C5 (2014–215), respectively (Fig. 3b). Interestingly, as C4 decreased, C3 rose again in abundance, indicating that members of C3 and C5 can coexist to a larger extent with each other than with members of cohort C4, respectively. Cohort C2, on the other hand, was present throughout the entire observation period and showed seasonal dynamics with peaks in abundance in the spring (Fig. 3b). Overall, these dynamics show that seasonal variation as well as decrease in community similarity over time is driven by successional changes in defined subcommunities.

Among the measured factors, the dynamics of microbial cohorts were strongly linked to MCRT, dissolved oxygen (DO), pH, temperature, and mixed liquor suspended solids (MLSS), as shown in the dbRDA analysis (ANOVA *P* < 0.001, Supplementary Figure S9). The successional patterns among cohorts C3, C4, and C5 were mostly influenced by the increase in MCRT, overall explaining 33.9% of the total variation. Temperature, pH, and DO collectively explained 13.2% of the total variation and could be further linked to the differentiation of C2, C3, C4, and C5. For instance, high DO concentration in the aeration tank correlated with the bloom of C2, while higher temperatures and pH may have enriched cohort C5.

### Functional stability persists while taxonomic composition changes

**Fig. 4:**
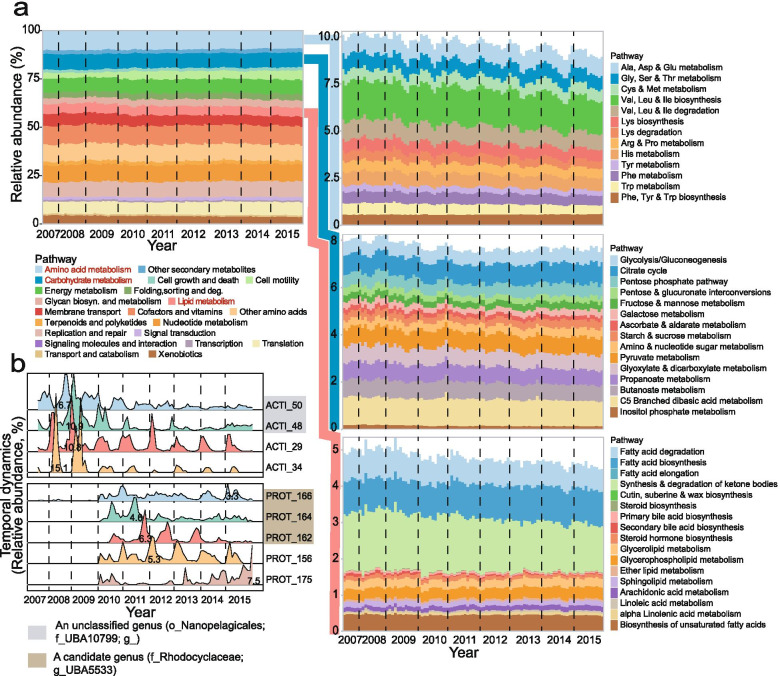
Microbial diversity contributes to the relatively stable functional distribution. **a** Functional profile of Kyoto Encyclopedia of Genes and Genomes (KEGG) standard categories. For each functional pathway, the relative abundance was calculated as the sum of marker KOs’ coverage normalized by the number of KOs. Relative abundance of a given functional pathway was normalized by the sums of all studied pathways’ abundance. Details of amino acid, carbohydrate, and lipid metabolisms are expanded. **b** Ridge plot shows the most abundant bacteria that were affiliated with the phyla Actinobacteriota and Proteobacteria. The numbers in the ridge plot are the maximum relative abundance of bacteria

In spite of the strong shift in taxonomic composition associated with the disturbance at the end of 2009, metabolic profiles proved remarkably stable throughout the entire 9-year sampling period (Fig. 4a and Supplementary Table S8). Key metabolic functions, such as amino acid, carbohydrate, lipid metabolisms, and energy generation remained nearly invariant before and after the bleaching event at the end of 2009. Taking energy metabolism as an example (Supplementary Figure S10), even level-3 pathways were highly invariant as evidenced by gene abundance of carbon fixation in photosynthetic organisms. This function only slightly increased from an average of 1.6% (2007–2009) to 1.7% (2010–2015) even though there was taxonomic turnover.

Consistent with the community-wide pattern, the two dominant bacterial phyla prior and after the disturbance also displayed high functional similarity. Principal coordinate analysis based on the KEGG metabolic modules of the key metabolic pathways revealed high overlap between Actinobacteriota and Proteobacteria, which dominated in the first and second community states, respectively (Supplementary Figure S11). Detailed analysis of the most abundant MAG clusters further confirmed the functional redundancy among taxonomically distinct microorganisms (Supplementary Figure S12). Metabolic modules involved in lipid, amino acid, and carbohydrate metabolisms that may affect the wastewater treatment process displayed high redundancy in the most abundant organisms prior (ACTI_48 and ACTI_50) and after (PROT_156, PROT_162, and PROT_164) the bleaching event. The overall functional stability in terms of carbon metabolisms was also confirmed by the relatively stable COD removal from the wastewater over 9 years (Supplementary Figure S13).

Interestingly, within the phyla Actinobacteriota and Proteobacteria, many of the most abundant MAG clusters showed repeated cycles of non-overlapping occurrence (Fig. 4b), suggesting that in addition to positive correlations analyzed above, more fine-scale succession and/or competitive exclusion also occur. Although these dynamics point to ecological differentiation among these very closely related MAGs, metabolic analysis confirms their high functional similarity. This pattern is consistent with recent findings that ecological differentiation of populations may involve few genes and may thus not be reflected in the relatively coarse-grained metabolic analysis (e.g., reconstruction of high-level metabolic pathways) that is usually used to judge functional similarity [59, 60]. Taken together, although the stability of key functions transcended the major shift in microbial community composition induced by the bleach addition in 2009, fine-scale dynamics show that there may be important differences that influence the dynamics of the community.

### Variation in function differentiates cohort populations

Considering the successional dynamics among microbial cohorts, we further asked what kind of functions might be variable over time amidst the overall stability. This analysis suggested that functional variation could be differentiated into continuously changing and cohort enriched pathways. Examples for continuous change are total gene abundances of cell motility, which increased from an average of 2.7% (2007–2009) to 4.3% (2010–2015) (Supplementary Figure S14). The increased prevalence of chemotaxis and flagellar assembly genes coincided with increasing MCRT and MLSS in the aeration tank after the end of 2010 (Supplementary Table S2). In addition, total genes indicative of nitrification continuously increased even though the KEGG nitrogen metabolism was highly stable. This change in nitrification was accompanied by increasing MCRT (Supplementary Figure S10) and was consistent with the more stable ammonia removal performance (Supplementary Figure S15), suggesting that the low growth rate organisms, such as ammonia oxidizers and nitrite oxidizers, were favored by the longer MCRT.

Five putative polyphosphate accumulating organisms (PAOs), three *Candidatus* Accumulibacter MAGs (PROT_157, PROT_158, and PROT_159) and two *Tetrasphaera elongate* MAGs (ACTI_29 and ACTI_30), were identified from the AS metagenomes. ACTI_29 affiliating with cohort C2 was identified as seasonal prevalent PAO with relative abundance up to 10.9% (December 15, 2008) (Fig. 4b). Controlling phosphorous discharged from Shatin WWTP is not a key factor in preventing eutrophication of seawater. Therefore, the system was not deliberately operated to achieve biological removal of phosphorous, which may explain the observed weak correlations between ACTI_29 and phosphorous removal (Spearman’s *ρ* = 0.37*,* adj. *P* < 0.001). Additionally, a *Gordonia* bacterium (ACTI_34) affiliating with cohort C2 known to cause foaming was significantly inhibited after the end of 2009 (Fig. 4b), which was in line with the mitigation of sludge foaming after the bleach addition.

**Fig. 5:**
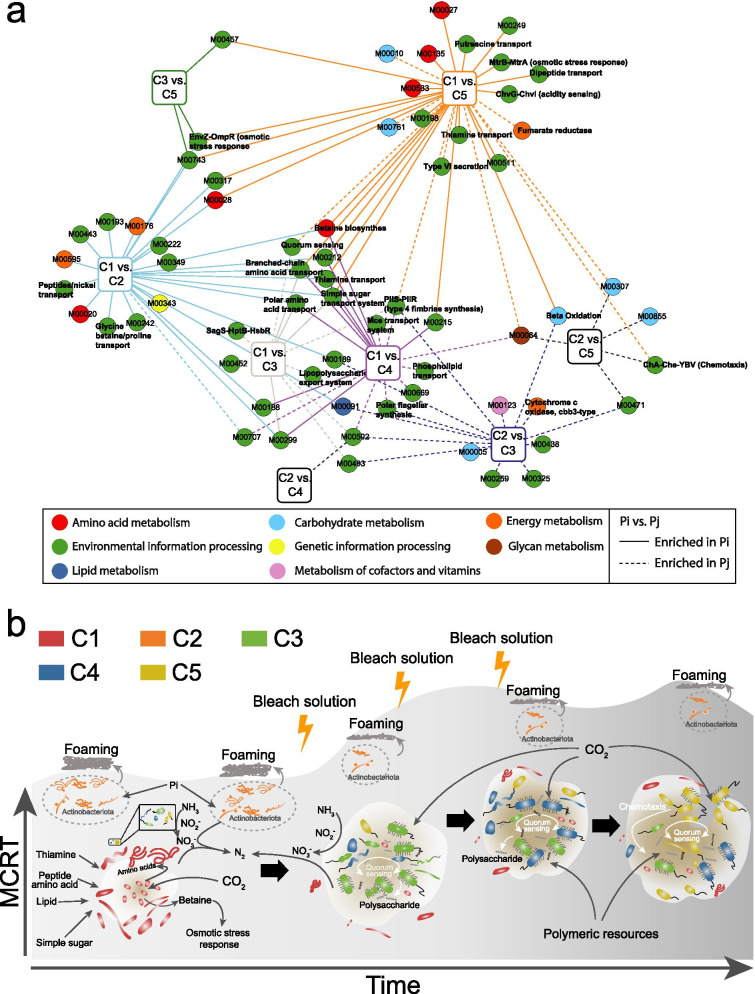
Comparative genomic analysis. **a** Pairwise comparison of enriched metabolic modules between two microbial cohorts. Each circle node represents > 60% steps/reactions of a specific metabolic module that are significantly enriched in given microbial cohort. Node color represents different standard KEGG categories. The solid line and dash line represent metabolic modules enriched in which cohort when conducting pairwise comparison. Details of enriched modules can be found in Supplementary Table S9. **b** Schematic overview of microbial dynamics and morphological changes over time. Microorganisms from different cohorts are represented with different colors. The morphological change of sludge over time was predicted based on comparative genomic analysis

Although cohorts were highly similar in central carbon and energy metabolisms, individual microorganisms from different cohorts did indeed show significant differentiation in a few metabolic as well as environmental information processing modules, classified as membrane transport, cell motility, and cellular community (e.g., quorum sensing and biofilm formation). These significantly enriched (adj. *P* value cutoff of 0.05) KEGG modules were identified by pairwise comparison of all cohorts based on the annotation results (KO matrix) of microbial MAG clusters (Fig. 5a, Supplementary Table S9). Accordingly, MAGs from cohort C1 showed significant (adj. *P* < 0.05) enrichment of the dipeptide, branched-chain amino acids, polar amino acid, thiamine, simple sugar transportation systems, suggesting higher reliance on peptides and amino acids as carbon sources. Given the unique saline sewage of the studied WWTP, enrichment of osmolarity response modules (e.g., betaine biosynthesis, osmotic stress response, and acidity sensing) in individual organisms of C1 was consistent with the more intensive fluctuation and higher maximum concentration of salinity in the first 5 years (2007–2011) than in the following years (Supplementary Figure S16).

Enrichment of KEGG modules also suggested increased importance of biofilm formation in the microorganisms affiliating with the successional cohorts C3-C5. For example, type IV fimbriae synthesis was significantly enriched (*P* < 0.05) in the individual members of these cohorts (i.e., C3-C5), indicating more widespread ability to attach to surfaces. Additionally, MAGs within C3 and C4 harbored significantly more genes for quorum sensing, which are important for communication in biofilms at high cell density [61]. Several functional modules, including swarming activity, biofilm formation, lipopolysaccharide export, and alginate production, were more prevalent (*P* < 0.05) in C3 compared to C1, revealing an increased ability to aggregate in the cohort that was dominant after the bleaching event (Fig. 5b). Finally, increasing biomass concentration in the AS system coincided with a higher relative gene copy number of glycoside hydrolases (GH) in C4 and C5 differentiating them from the cohorts dominating in the first 5 years (Supplementary Figure S17). Specifically, genes encoding endoglucanase (GH74) exhibited at much higher frequency in C4 and C5, and genes for cellulose binding were twofold more abundant in the other cohorts. Thus, along with increased ability to attach, a shift from reliance on simple sugar to polymeric resources (i.e., solid), may have occurred within the second community state under increasing MCRT and increased ability to aggregate (Fig. 5b).

## Discussion

How taxonomic structure relates to functional performance of microbial communities is a key question in microbial ecology. Our long-term longitudinal sampling of self-assembled communities in a WWTP suggests that the highly similar environmental conditions can support divergent communities that remain relatively stable over time and perform the same essential cellular functions. A simple, short-term disturbance induced the shift between these alternative stable states where the chlorination event appears to have affected the originally dominant Actinobacteriota disproportionately, leading to their replacement of functionally highly equivalent Proteobacteria. Moreover, the abrupt shift of taxonomic composition after the end of 2009 could explain our previously observed viral dynamics pattern that only a few of the viral populations recovered from samples taken in 2016 were detected before 2010 [26]. This dynamic is also a rare example of the original concept proposed by Lewontin that communities can exist in different stable states under fixed environmental conditions and that disturbances specifically affecting some populations can induce transitions between these states [62]. Similar dynamics have been observed in gut microbiomes where disturbances such as major diarrheal events can induce taxonomically distinct communities although the functional components remain less explored [63]. More common, however, is the observation that changes in environmental parameters can induce shifts to communities that are also functionally different [1].

Despite the observed abrupt shifts in microbial community compositions, key metabolic functions remained remarkably stable over time. Such functional redundancy has been reported from other systems, including water collecting in *Bromeliads* [2], spatially separate ocean samples [64], subseafloor aquifer [65], and human microbiomes [66]. However, in most cases, the environmental conditions significantly varied across spatially distinct samples and it remains unclear whether more subtle, unmeasured factors induce assembly of alternative communities under a fixed condition. In other words, would different community states be stable under the observed spatial and/or temporal manifestations of the habitat type? Here, longitudinal sampling was applied to an aeration tank of a full-scale WWTP that remained under highly similar operational conditions in the first 4 years (2007–2010), i.e., transcending disturbance-induced community shift at the end of 2009. This strongly suggests that the overall communities are truly functionally equivalent and can stably replace each other. The data indicate that functions were highly preserved in this period, even if some traits may be distributed in different combinations across taxa. Indeed, while the dominant MAGs that replaced each other displayed high similarity, there were differences in genetic endowment suggesting that some functions might be encoded in other community members. Such differential distribution of traits among members of different communities may create different ecological dependencies, for example, via community-specific patterns of cross-feeding. Specificity of interactions may provide cohesion to communities and prevent members from functionally equivalent communities from invading, ensuring stability of the community. It should be noted that the functional profile estimated based on gene and MAGs relative abundance cannot be directly linked to metabolic activities, but comparative metatranscriptomics is beyond the scope of the present study.

In support of the notion that biological interactions may stabilize communities, our analysis of the dynamics also revealed correlated cohorts of MAGs that display successional dynamics on both seasonal and longer time scales. Such dynamics can be deduced from longitudinal sampling allowing interpretation of community responses in light of changes in environmental factors [58, 67, 68]. That the cohorts in the latter observation period (after 2010) when operating conditions changed gradually also transitioned in rather abrupt fashion suggests that communities were resilient toward disturbance [69, 70], i.e., they did not change markedly until a threshold was reached. In the case of the WWTP, the dominant factor was most likely the increase in MCRT, which also led to the most pronounced shift in functional properties in the communities. Later cohorts displayed more potential to aggregate, form biofilms and digest polymers, while the earliest cohort was enriched in genes suggestive of metabolism of labile compounds. This shift is consistent with the increased importance of solid resources induced by the higher residence time in the aeration tank. Moreover, the increased ability to aggregate in later cohorts may contribute to community tolerance or resistance of subsequent bleaching events after the end of 2009 [71, 72]. Seasonal disturbances, on the other hand, led to the regular expansion of one cohort but the community essentially fluctuated around an average, meaning it displayed resilience toward seasonal factors such as shifts in temperature. Finally, resilience also suggests that changes in operational parameters might be titrated to determine the tipping point of community change, especially if one community state proves superior to another in performance.

## Conclusions

Alternative stable states in communities are key concepts in environmental management and engineering [73]. Our long-term longitudinal sampling and metagenome analysis of AS from a full-scale WWTP confirmed that the highly similar environmental conditions can support divergent communities that remain relatively stable over time and perform the same essential cellular functions. The identification of abrupt shifts in microbial community compositions induced by a simple, short-term disturbance may also help manage applied microbial systems such as WWTPs that are currently primarily monitored by taxonomic surveys. As the studied AS system is a unique saline ecosystem, important questions going forward will be how similar stable state communities identified in one system are to another. Resilience of communities may provide a window of conditions under which highly similar communities assemble. How narrow or wide such conditions may be, remains an open question but the remarkable scope of microbial diversity in nearly all environments [58, 74] suggests that microbes finely partition this environmental niche space.

## Supplementary Information

Supplementary file1 (DOCX 3274 KB)

Supplementary file2 (XLSX 890 KB)

## Data Availability

The raw nucleotide sequence data and MAGs used in the present study have been deposited in the NCBI database under project ID PRJNA432264.

## Abbreviations

ANI: Average nucleotide identity
AS: Activated sludge
dbRDA: Distance-based redundancy analysis
DO: Dissolved oxygen
eLSA: Extended local similarity analysis
LS: Local similarity
MAGs: Metagenome-assembled genomes
MCL: Markov clustering algorithm
MCRT: Mean cell residence time
MLSS: Mixed liquor suspended solids
ORFs: Open reading frames
PAOs: Polyphosphate accumulating organisms
WWTPs: Wastewater treatment plants
