# Supplementary material for: Successional dynamics and alternative stable states in a saline activated sludge microbial community over 9 years
Source: Microbiome. 2021 Oct 6;9:199. doi: 10.1186/s40168-021-01151-5 (PMC8495973; doi:10.1186/s40168-021-01151-5)
Supplement: Supplementary file 2 — Supplementary file1 (DOCX 3274 KB) [file 40168_2021_1151_MOESM1_ESM.docx]

**Supplementary Information**

**for**

**Successional dynamics and alternative stable states in a saline activated sludge microbial community over 9 years**

Yulin Wang^1^, Jun Ye^2^, Feng Ju^3^, Lei Liu^1^, Yu Deng^1^, Joel A. Boyd^2^, Donovan H. Parks^2^, Xiaotao Jiang^1^, Xiaole Yin^1^, Ben J. Woodcroft^2^, Gene W. Tyson^2^, Philip Hugenholtz^2^, Martin F. Polz^4,5^ and Tong Zhang^1*^

^1^ Environmental Microbiome Engineering and Biotechnology Laboratory, The University of Hong Kong, Hong Kong SAR, China.

^2^ Australian Centre for Ecogenomics, School of Chemistry and Molecular Biosciences, The University of Queensland, Brisbane, Queensland, Australia.

^3^ School of Engineering, Westlake University, 18 Shilongshan Road, Hangzhou 310024, China.

^4^ Department of Civil and Environmental Engineering, Massachusetts Institute of Technology, Cambridge, MA 02139, USA.

^5^ Division of Microbial Ecology, Centre for Microbiology and Environmental Systems Science, University of Vienna, Vienna, Austria.

**Supporting Information S1: Used scripts in the present study.**

1. Script 1: Rarefaction curve of detected genes

CoverM (https://github.com/wwood/CoverM) was used to estimate the coverage values of genes in the reconstructed gene catalog among different AS metagenomic data. A python script deposited in GitHub (https://github.com/yulinwang605/identified-genes-in-randomly-samples) was used to summarize the number of identified genes in given number of randomly selected AS metagenomic datasets.

2. Script 2: Bray-Curtis dissimilarity analysis

The R script for pairwise Bray-Curtis dissimilarity was deposited in GitHub (https://github.com/yulinwang605/Pairwise-Bray-Curtis-dissimilarity/tree/main).

3. Script 3: Carbohydrate-active enzymes annotation

Genes coding for carbohydrate-active enzymes (CAZy) in each metagenome-assembled genomes (MAGs) was analyzed using the following script deposited in GitHub (https://github.com/yuboer/genome-centric-portrait-of-cellulose-hydrolysis) that developed by our team member.


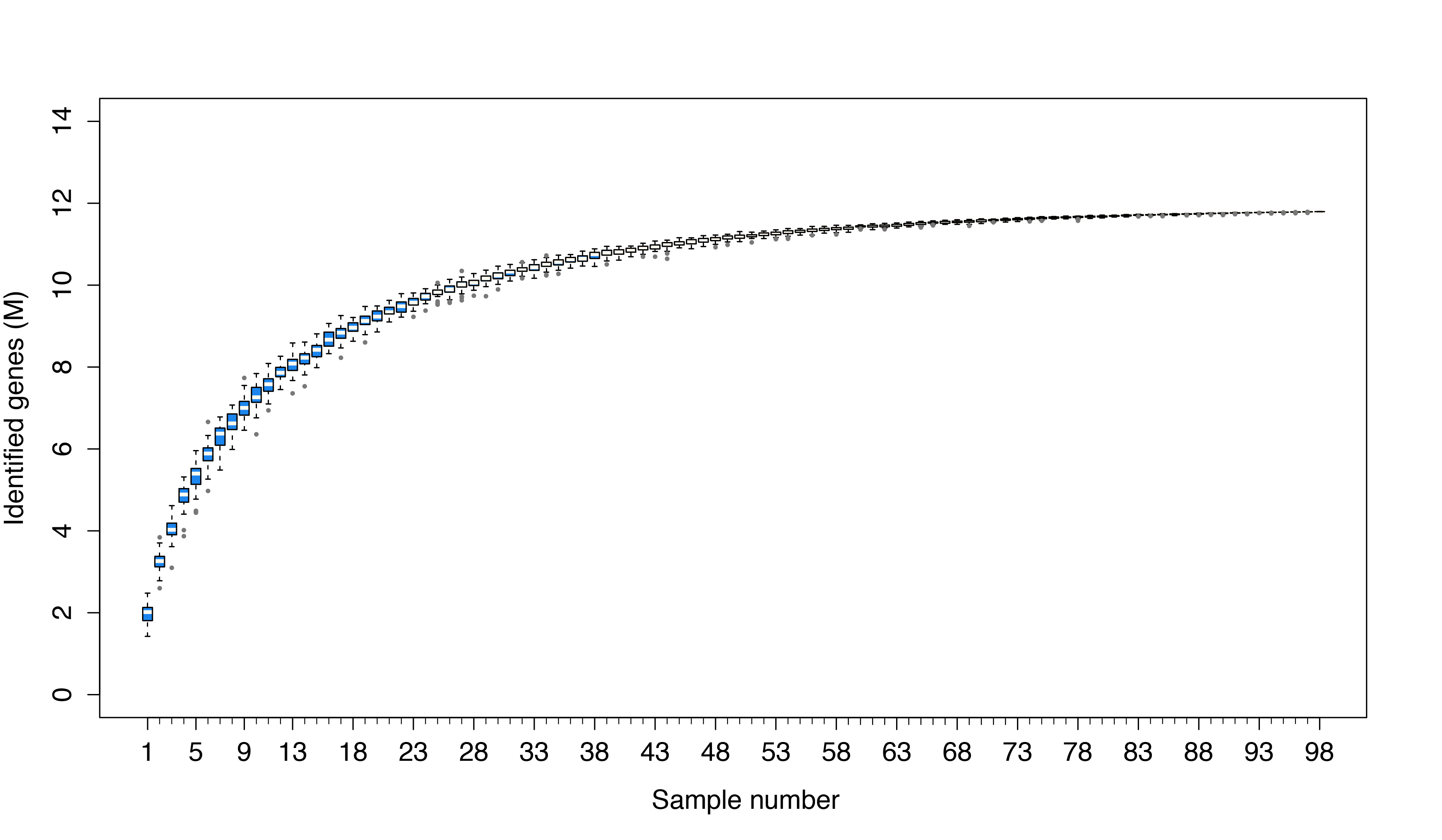


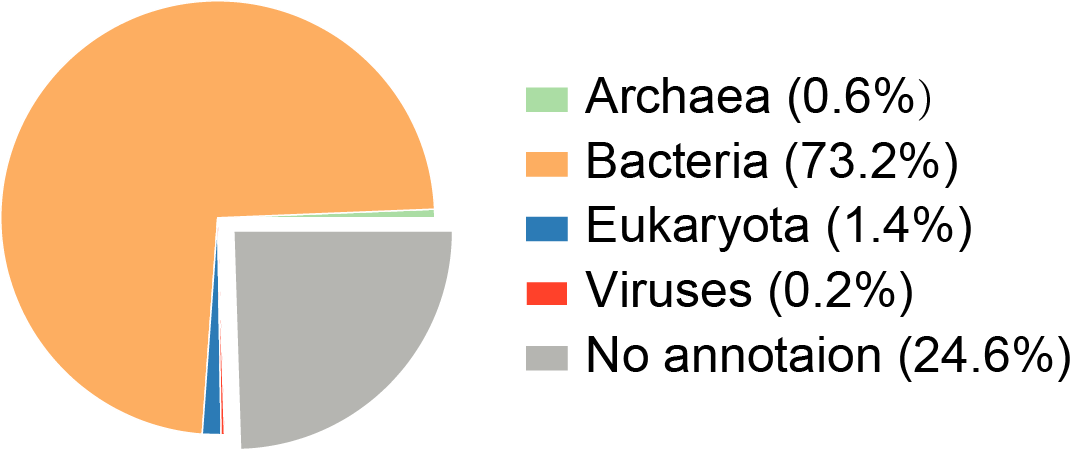


**Figure S1. Rarefaction curve of detected genes based on 100 permutations.** The gene that has a coverage >0.5 is considered to be detected in given sample. The pie chart shows the breakdown of taxonomic annotations at domain level.


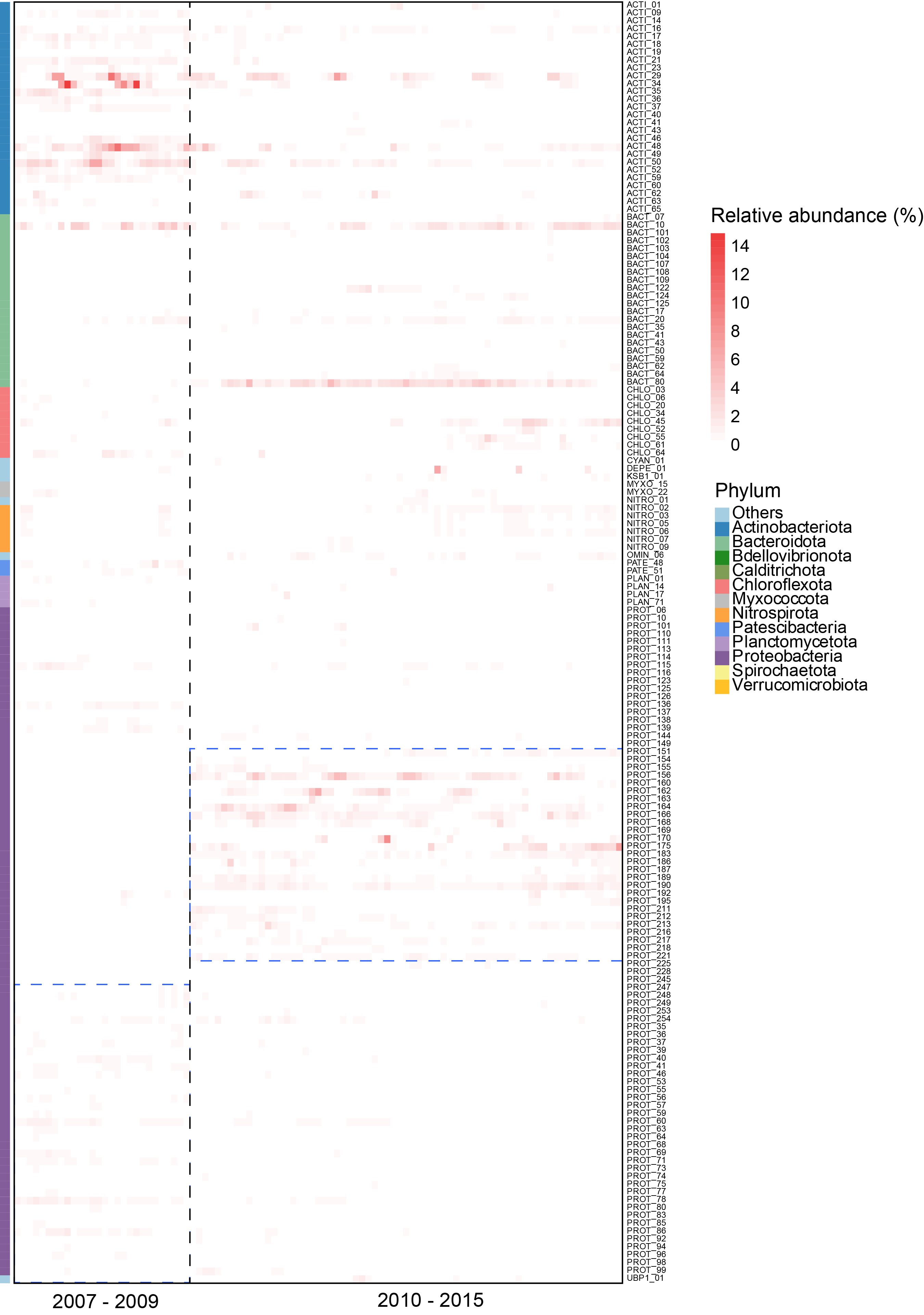


**Figure S2. Heatmap indicates the relative abundance of bacterial MAGs over nine years.** Only bacteria show an average abundance >0.1% were displayed in this heatmap. Phyla with average abundance < 0.5% are assigned into Others.


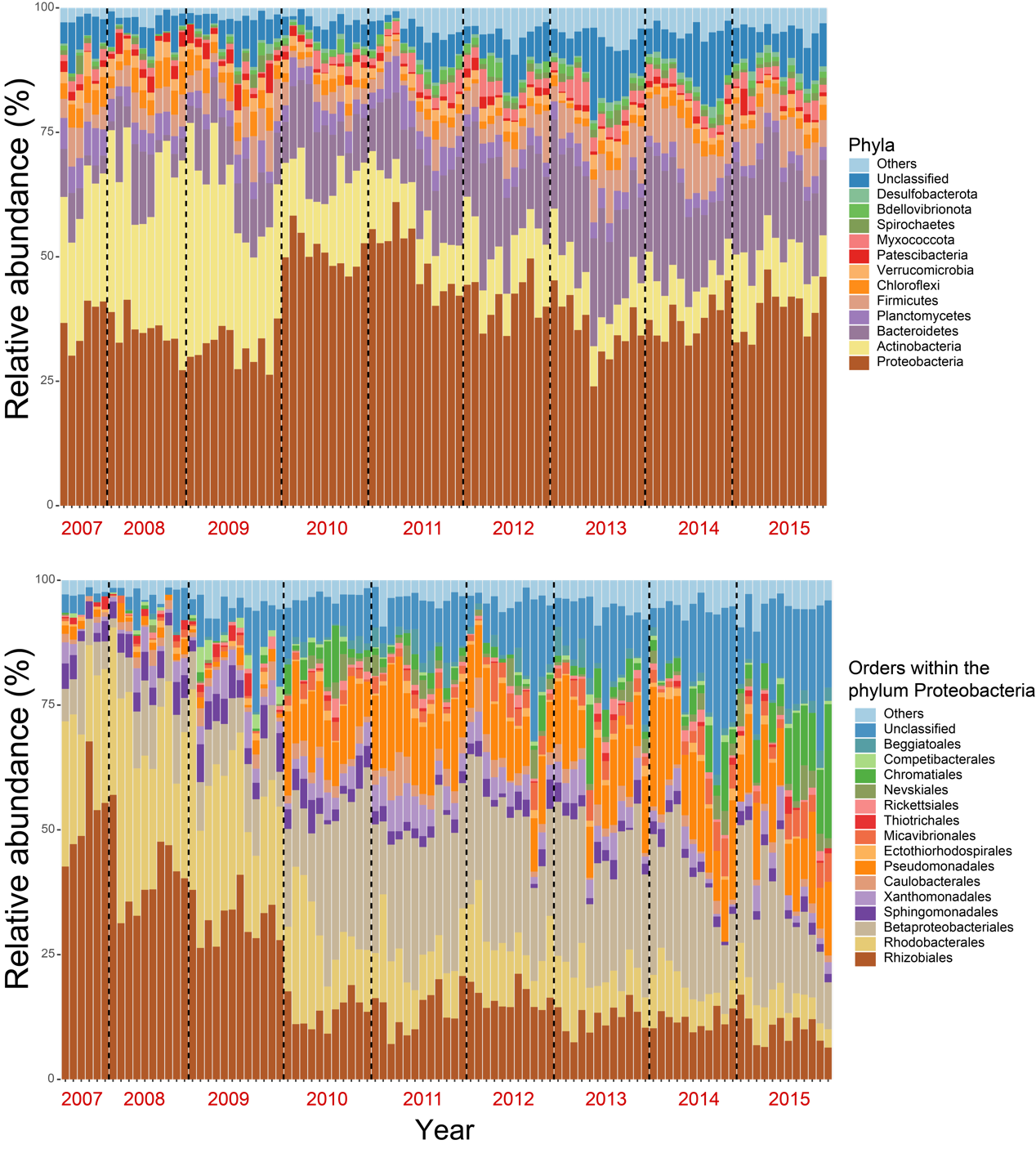


**Figure S3. Microbial community dynamics at phylum-level and order-level for Proteobacteria.** The abundance of microbial community was estimated using SingleM based on quality filtered metagenomic sequences.


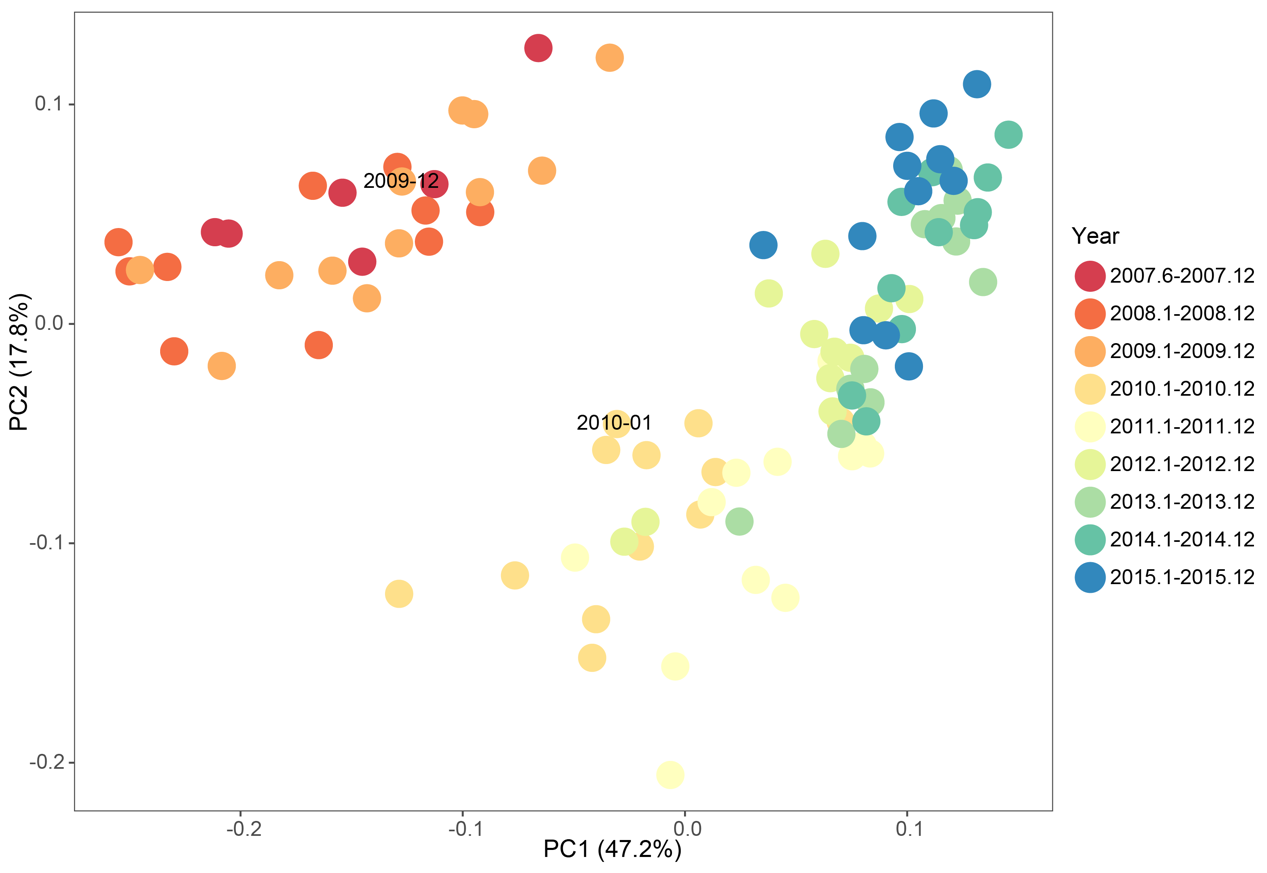


**Figure S4.** **Principal Coordinates Analysis (PCoA) of weighted Bray–Curtis community dissimilarity.** Community dissimilarity is estimated using the dynamics of microbial community at order level that was obtained from read-based results.


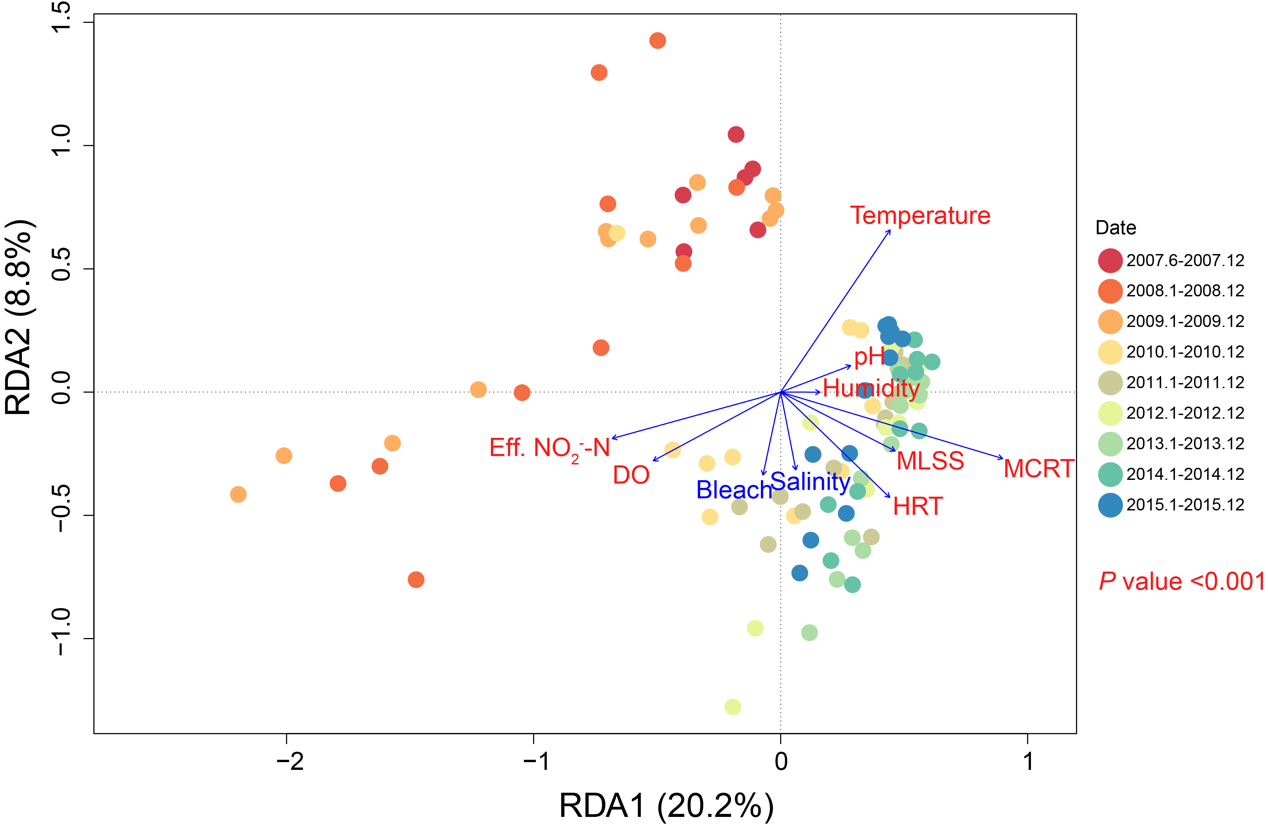


**Figure S5. Distance based redundancy analysis of the samples with the most explanatory variables.** Significant variables (ANOVA *P* <0.001) are in red. AS samples taken from different years are colored with different colors.


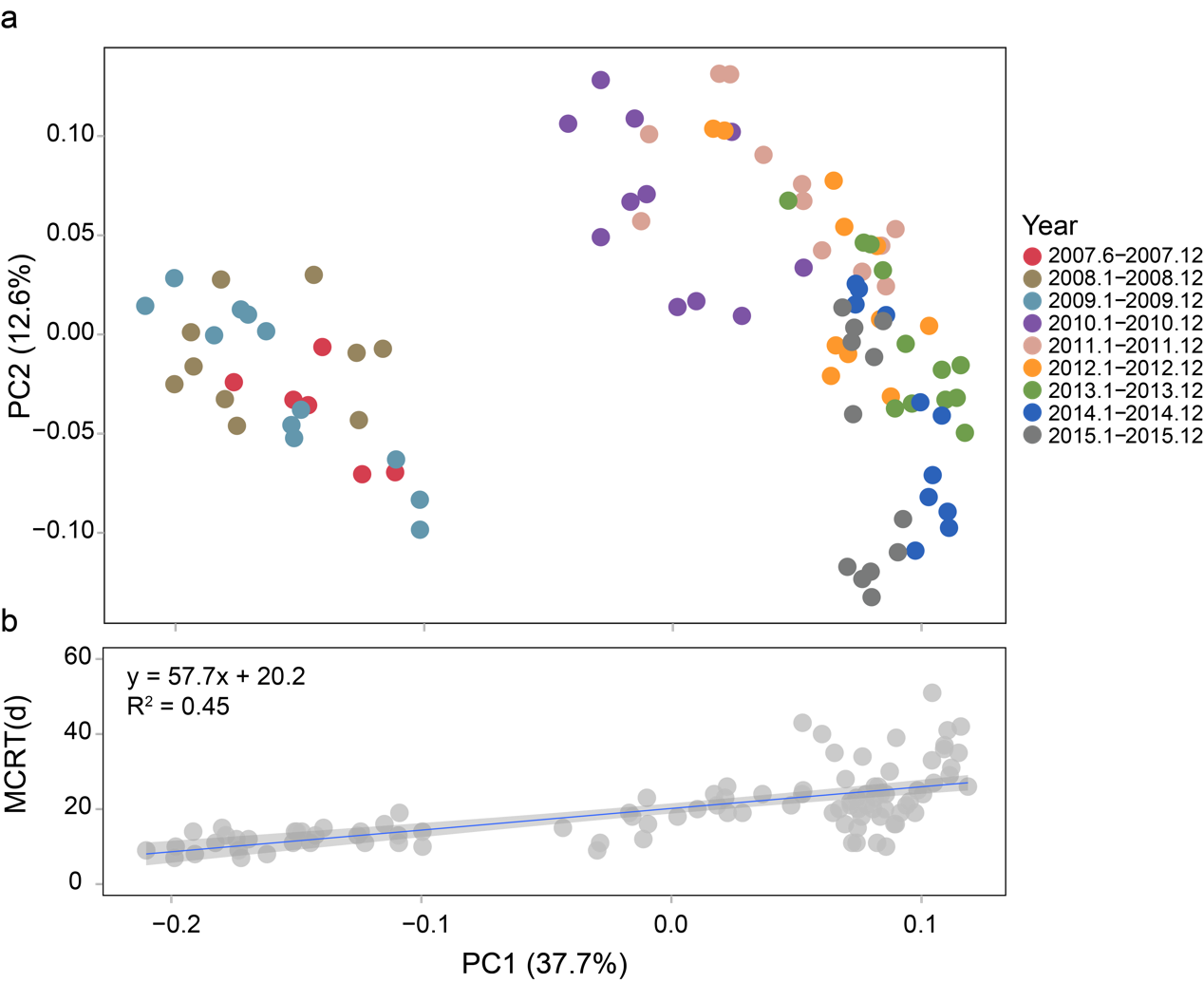


**Figure S6. Principal coordinate analysis (PCoA) of studied AS samples based on Bray-Curtis distances of MAGs-based community structure.** Scatter plot (b) shows correlation between the first PC and mean cell residence time (MCRT).


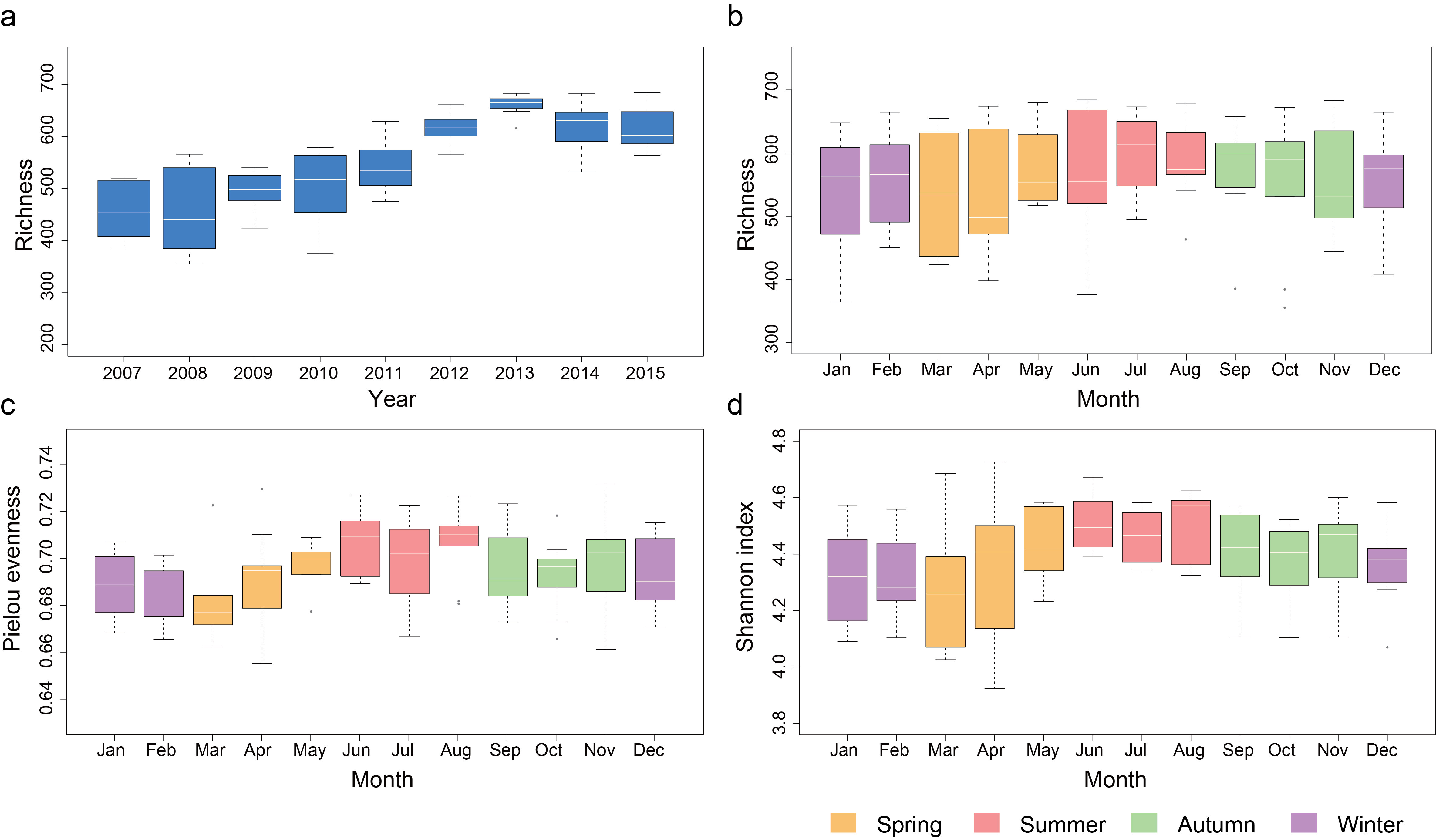


**Figure S7. Alpha-diversity distribution of the microbial community (MAGs-based) in AS system.** Colors in b, c, and d represent different seasons in Hong Kong.

**
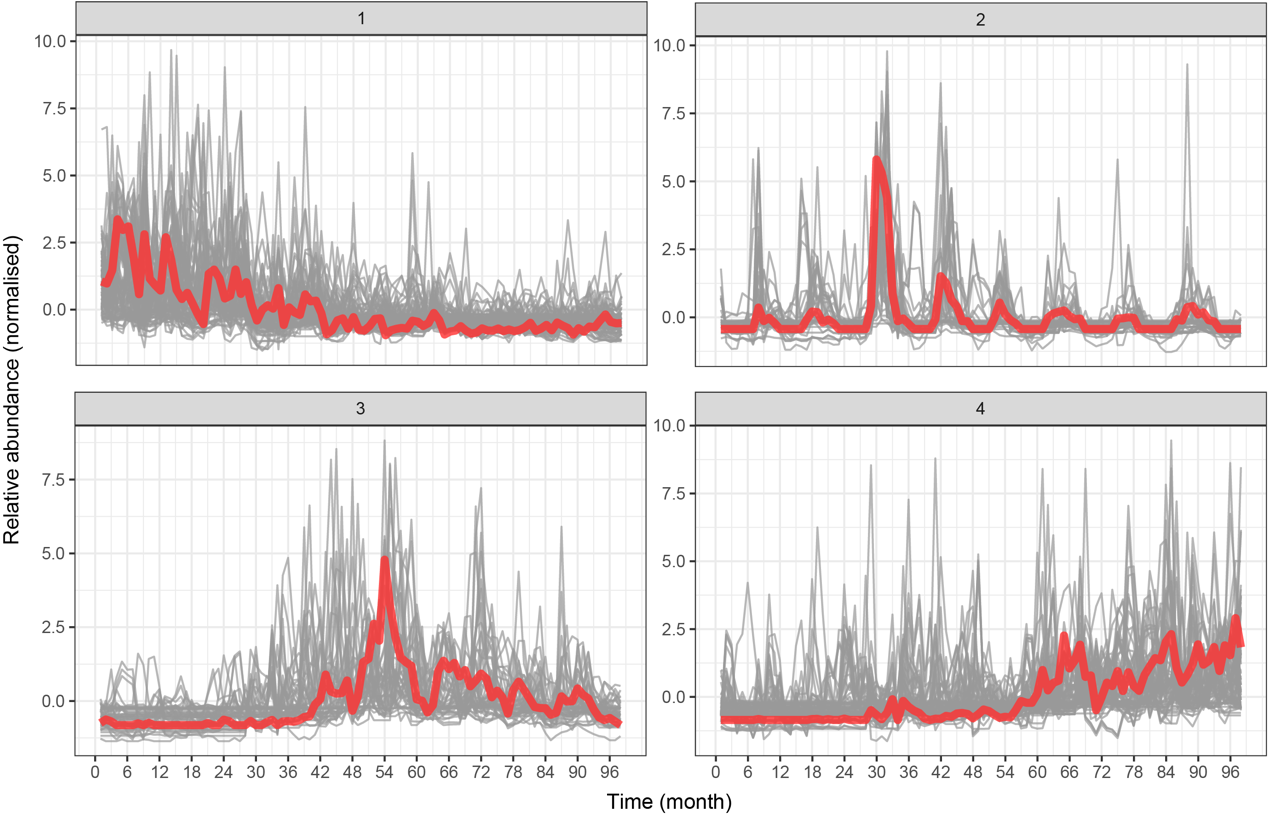
**

**Figure S8. Partitioning around medoids analysis algorithm for the microbial community members clustering.** Four clusters were specified before the execution of the algorithm according to the Davies–Bouldin index (DBI). Red lines represent the medoids of clusters.


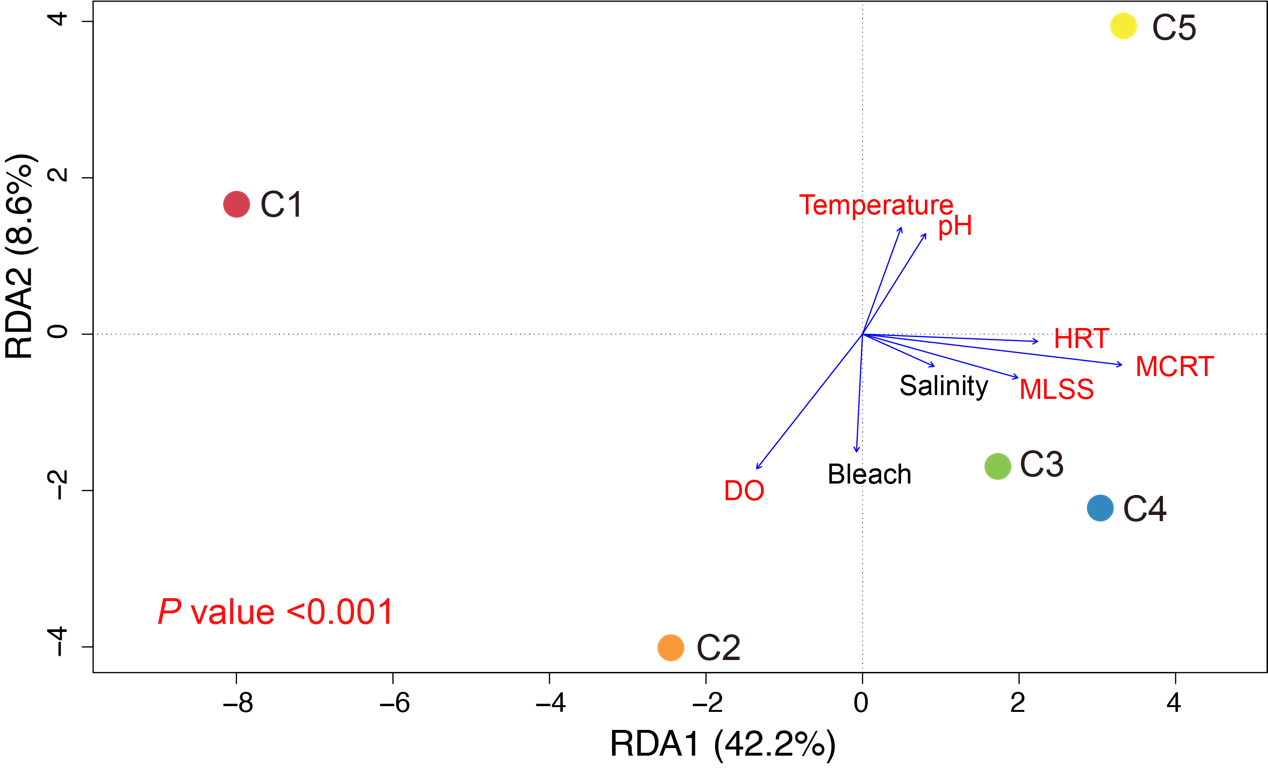


**Figure S9. Distance-based redundancy analysis (****dbRDA) of the microbial cohorts (dots).** Eight explanatory variables (arrows) best explained the dynamic patterns of these five cohorts with multivariate non-parametric ANOVA for selection of these significant variables (*P* <0.01). The parameters colored in red represent the *P* <0.001.


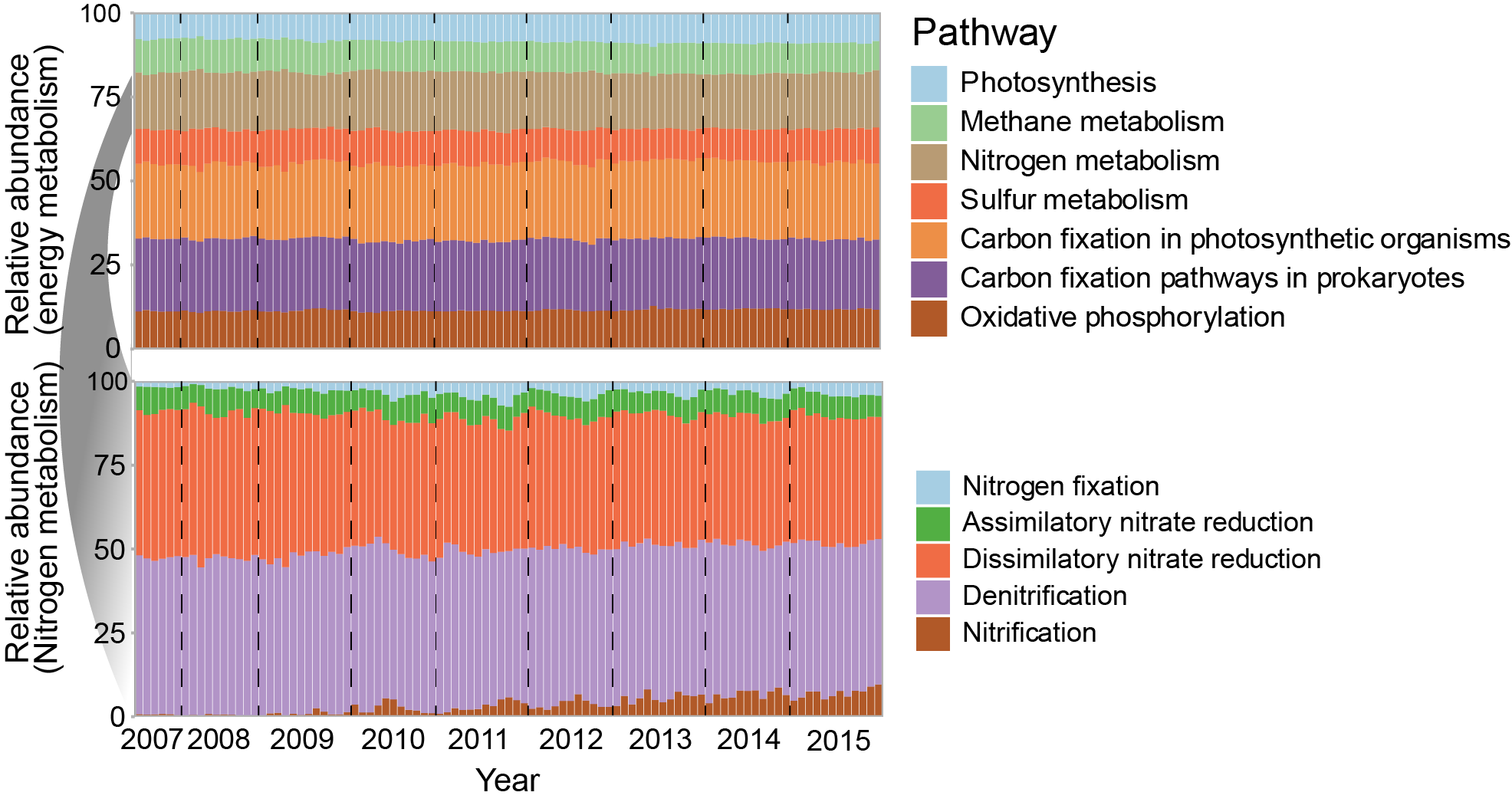


**Figure S10. Functional profile of Kyoto Encyclopedia of Genes and Genomes (KEGG) standard categories.** For each functional pathway, the abundance was calculated as the sum of marker KOs’ coverage normalized by the number of KOs. Relative abundance of a given functional pathway was normalized by the sums of all studied pathways’ abundance. The profile was characterized using the constructed non-redundant gene catalog instead of genes predicted from MAGs.


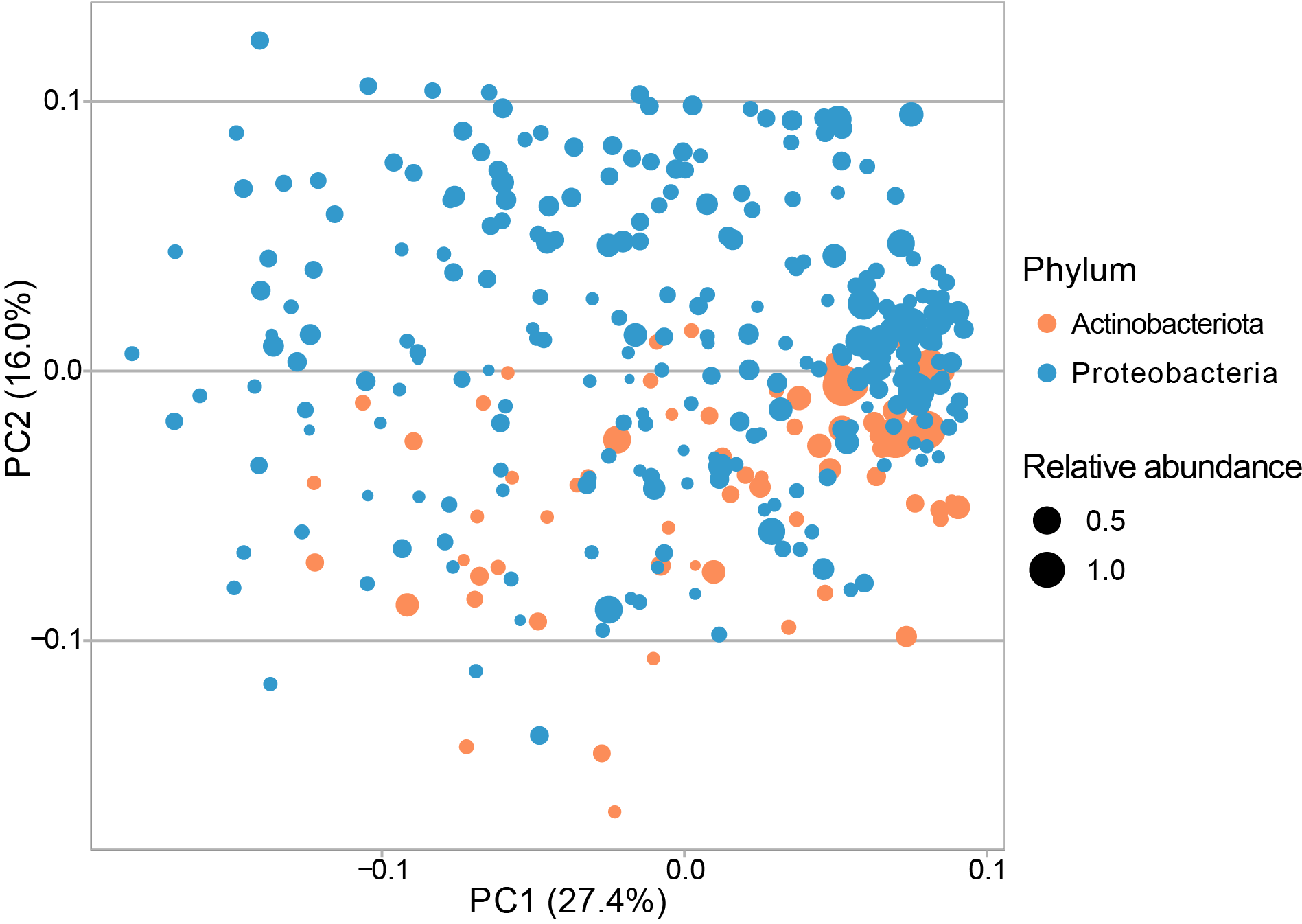


**Figure S11. Metabolic potential comparison of the abundant (relative abundance >0.5% at least once in the time series) bacteria within Actinobacteriota and Proteobacteria.** Principal coordinate analysis (PCoA) of the abundant bacteria is performed based on the matrix of key Kyoto Encyclopedia of Genes and Genomes (KEGG) metabolic modules (1 represents complete module with completeness ≥60% and 0 represents incomplete module) of central carbohydrate, energy, amino acid, lipid, fatty acid, cofactor and vitamin metabolisms. The size and color show the average abundance of MAG clusters and their taxonomic affiliation, respectively.


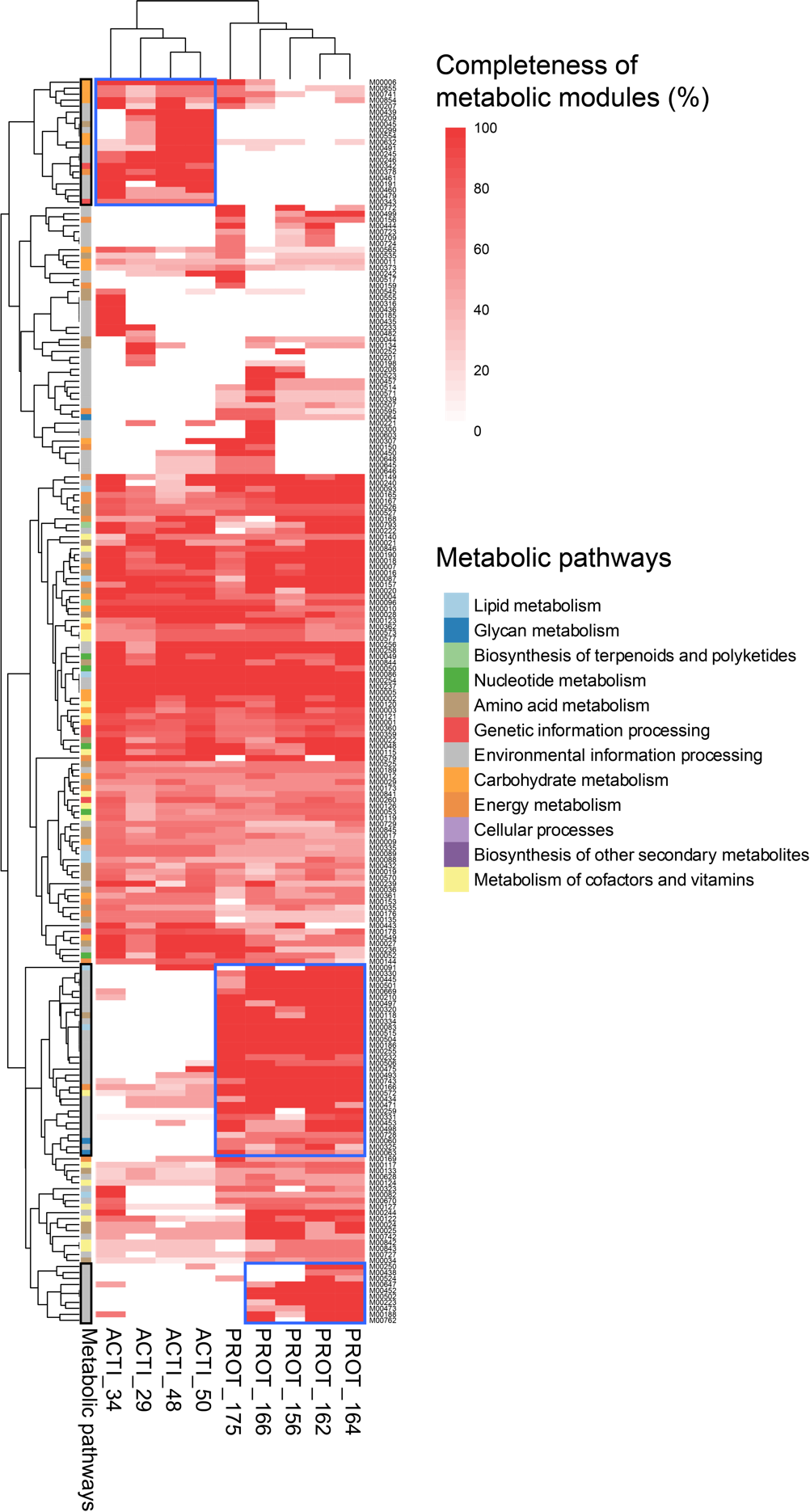


**Figure S12. Completeness of metabolic modules of the most abundant bacteria.** Only modules that show completeness >60% in at least one bacterium are displayed here.


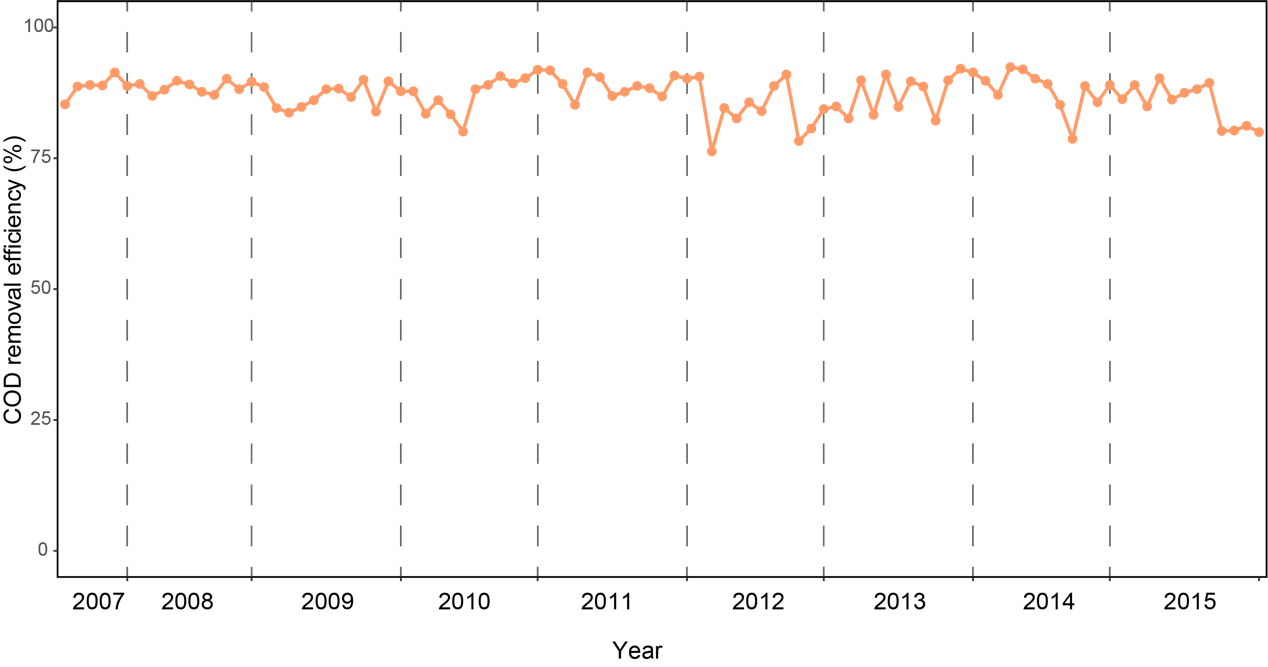


**Figure S13. COD removal efficiency over the nine years.**

**Figure S14. Dynamics of metabolic pathways involved cell mobility (a) and fold change between bacterial chemotaxis and flagellar assembly over the nine years (b).** The profile was characterized using the constructed non-redundant gene catalog instead of genes predicted from MAGs.


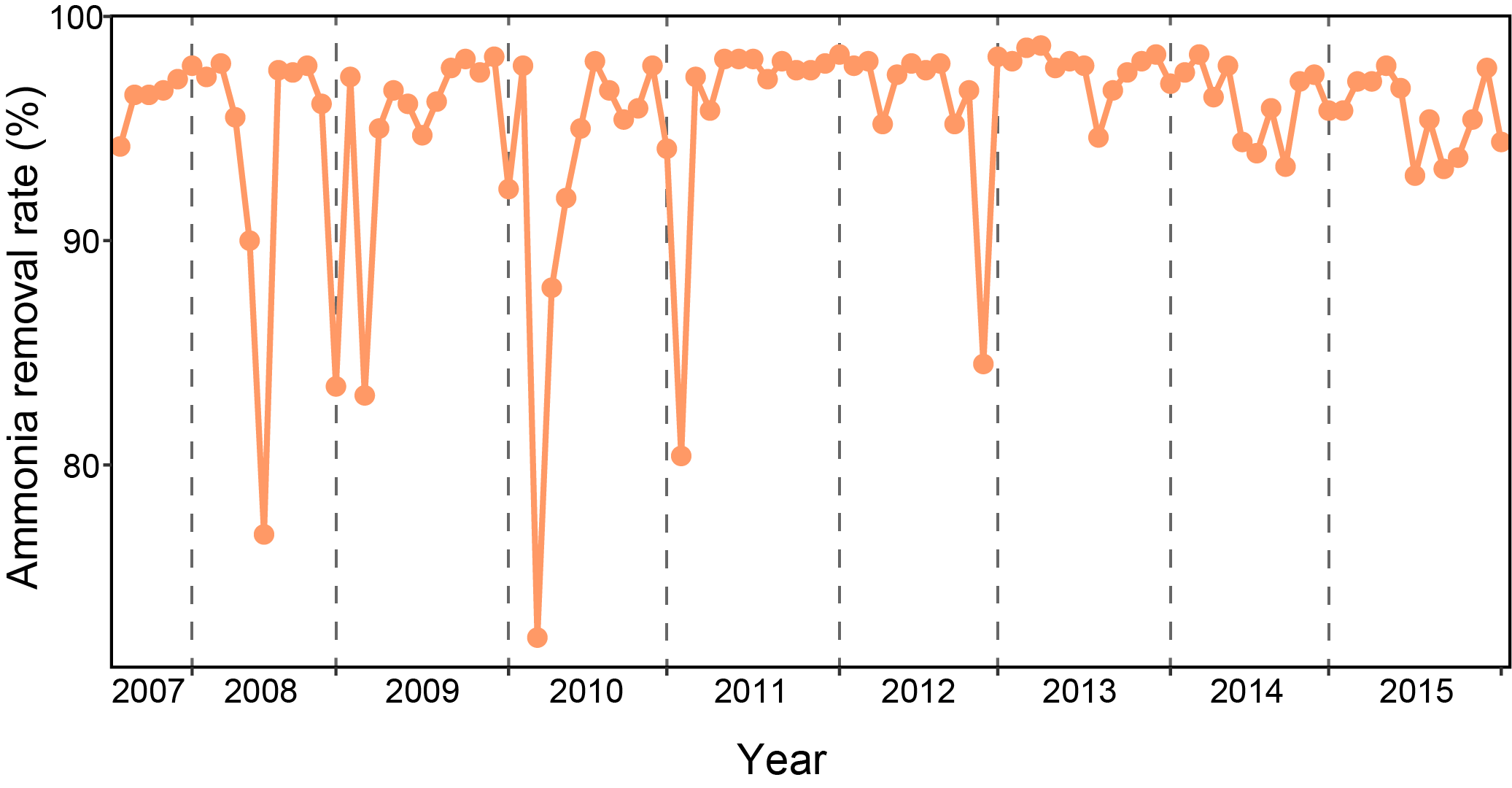
**Figure S15. Ammonia removal rate over the nine years.**


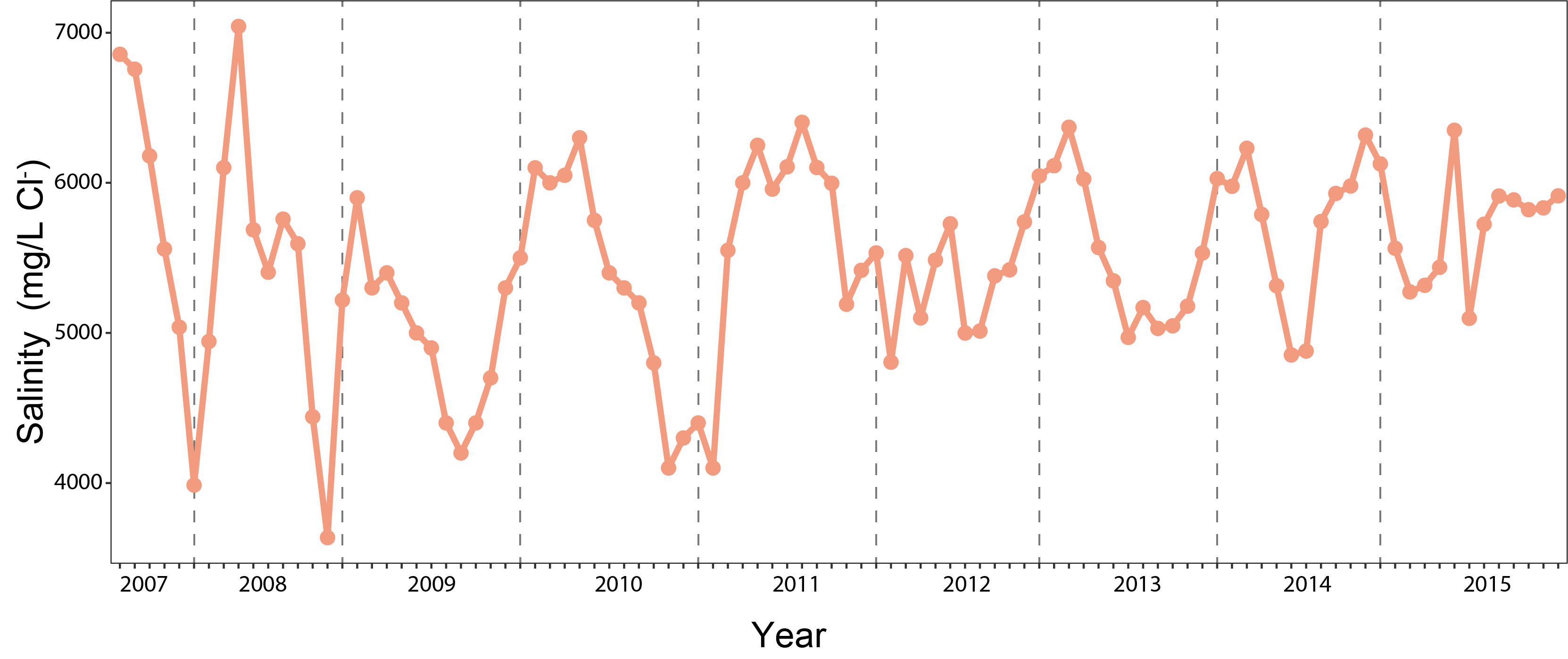


**Figure S16. Changes of salinity of the sewage in Shatin WWTP measured by mg/L Cl^-^.**


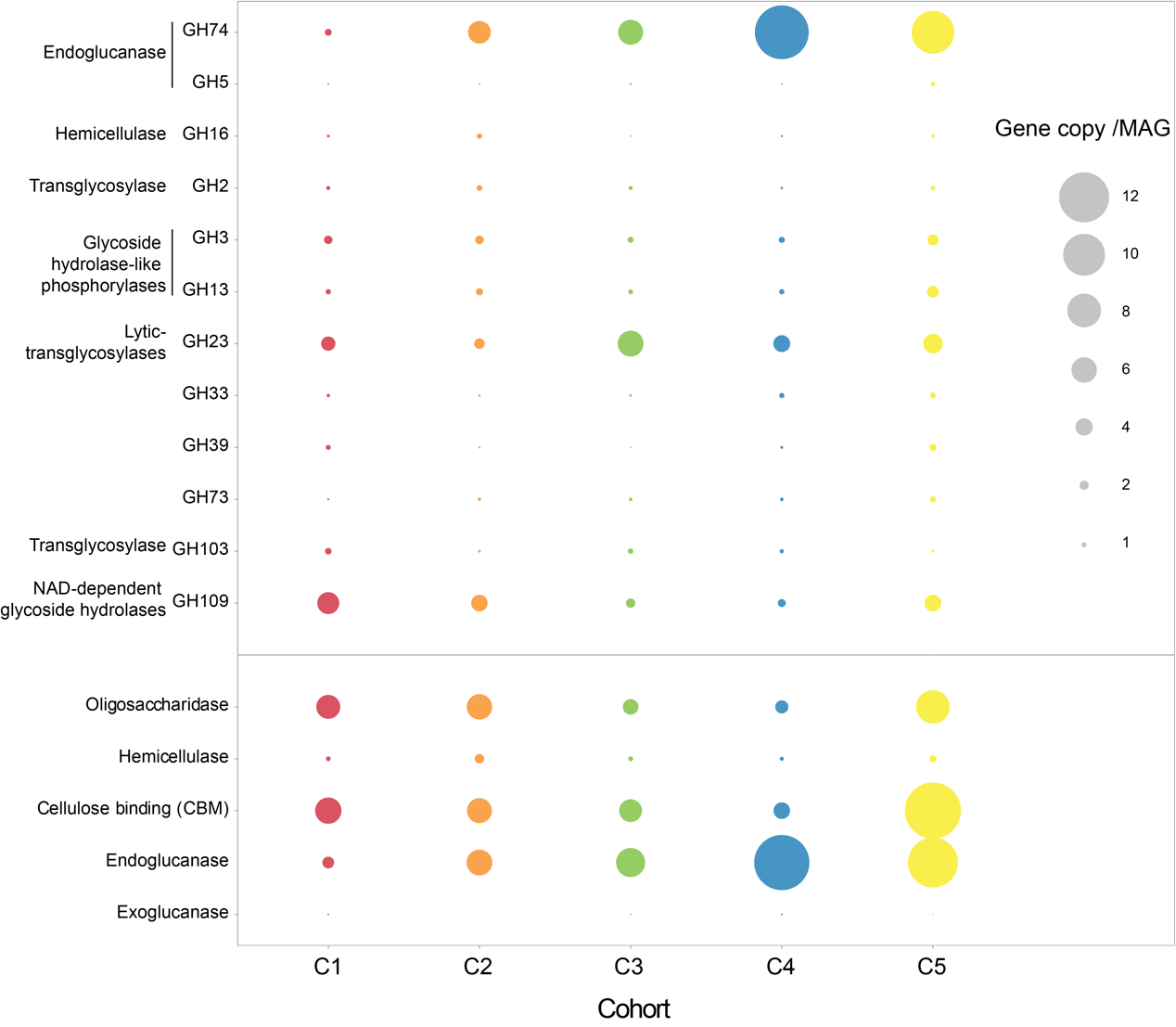


**Figure S17. Relative abundance of genes codding for carbohydrate-active enzymes (CAZy) in different microbial cohorts.** Bubble size represents the average copy number of GH gene in per metagenome-assembled genome (MAG). Abundant GH families are represented in the upper panel. The lower panel summarizes the overall functional groups of all CAZy modules.
